# Synthesis of Diversely Substituted Diethyl (Pyrrolidin-2-Yl)Phosphonates

**DOI:** 10.3390/molecules30092078

**Published:** 2025-05-07

**Authors:** Andrea Bagán, Alba López-Ruiz, Sònia Abás, Elies Molins, Belén Pérez, Itziar Muneta-Arrate, Luis F. Callado, Carmen Escolano

**Affiliations:** 1 Laboratory of Medicinal Chemistry, Department of Pharmacology, Toxicology and Medicinal Chemistry, Faculty of Pharmacy and Fo Sciences, University of Barcelona, Av. Joan XXIII, 27-31, 08028 Barcelona, Spain; andreabaganp@gmail.com (A.B.); alopezru@ub.edu (A.L.-R.); soniaabas88@gmail.com (S.A.); 2 Institute of Biomedicine, University of Barcelona, 08028 Barcelona, Spain; 3 Institut de Ciència de Materials de Barcelona (CSIC), Campus UAB, 08193 Cerdanyola del Vallés, Spain; elies.molins@icmab.es; 4 Department of Pharmacology, Therapeutic and Toxicology, Autonomous, University of Barcelona, 08193 Cerdanyola del Vallés, Spain; belen.perez@uab.cat; 5 Department of Pharmacology, University of the Basque Country (UPV/EHU), 48940 Leioa, Bizkaia, Spain; itziar.muneta@ehu.eus (I.M.-A.); lfcallado@ehu.eus (L.F.C.); 6 Centro de Investigación Biomédica en Red de Salud Mental, CIBERSAM, 28029 Madrid, Spain; 7 BioBizkaia Health Research Institute, 48903 Barakaldo, Spain

**Keywords:** α-aminophosphonate, pyrroline, phosphonic ester, (pyrrolidine-2-yl)phosphonate, phosphoproline, imidazoline I_2_ receptor ligand

## Abstract

Imidazoline I_2_ receptors (I_2_-IR) are untapped therapeutic targets lacking a structural description. Although the levels of I_2_-IR are dysregulated in a plethora of illnesses, the arsenal of ligands that can modulate I_2_-IR is limited. In this framework, we have reported several new structural families embodying the iminophosphonate functional group that have an excellent affinity and selectivity for I_2_-IR, and selected members have demonstrated relevant pharmacological properties in murine models of neurodegeneration and Alzheimer’s disease. Starting with these iminophosphonates, we continued to exploit their high degree of functionalization through a short and efficient synthesis to access unprecedented 2,3-di, 2,2,3-tri, 2,3,4-tri, and 2,2,3,4-tetrasubstituted diethyl (pyrrolidine-2-yl) phosphonates. The stereochemistry of the new compounds was unequivocally characterized by X-ray crystallographic analyses. Two selected compounds with structural features shared with the starting products were pharmacologically evaluated, allowing us to deduce the required key structural motifs for biologically active aminophosphonate derivatives.

## 1. Introduction

**Figure 1:**
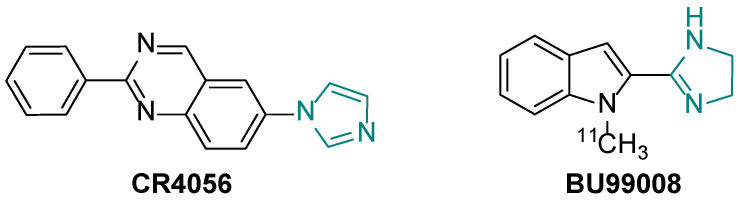
I_2_-IR ligands CR4056 and BU99008, with the imidazole/imidazoline nucleus in color.

We have previously reported that the reaction of diethyl isocyanomethylphosphonates and *N*-substituted maleimides in the presence of a catalytic amount of AgOAc underwent diastereoselective [3+2]cyclocondensation, leading to bicyclic α-iminophosphonates [1]. These new structures revealed bioactivity, showing a high affinity and selectivity for imidazoline I_2_ receptors (I_2_-IR) [2,3,4], being the first modulators of these receptors lacking an imidazole/imidazoline nucleus [1]. I_2_-IR are undescribed from the structural point of view, but have been validated as therapeutic targets, with the first-in-class I_2_-IR ligand, CR4056, progressing in a clinical phase II, multisite, randomized, placebo-controlled clinical trial for knee osteoarthritis patients (Figure 1) [5]. Also, [^11^C] BU99008, identified as an I_2_-IR ligand, is a clinical candidate in phase I for Positron Emission Tomography diagnostics in patients suffering from Parkinson’s disease and Alzheimer’s disease (AD) (Figure 1) [6].

**Scheme 1:**
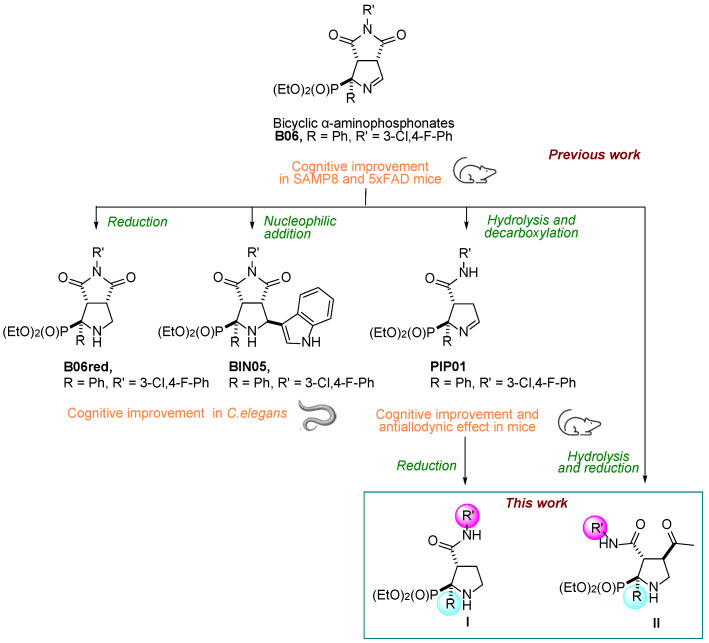
Outline of the previously reported work on representative B06, with the key reactions that led to access to B06red, BIN05, and PIP01 and significant in vivo results. General structures I and II of diversely substituted pyrrolidine-2-phosphonates reported in this work and key reactions.

Within our new non-imidazoline I_2_-IR ligands, a selected bicyclic α-iminophosphonate endowed with excellent affinity upon I_2_-IR, (1*RS*,3*aSR*,6*aSR*)-5-(3-chloro-4-fluorophenyl)-4,6-dioxo-1-phenyl-1,3a,4,5,6,6a-hexahydropyrrolo[3,4-*c*]pyrrole-1-phosphonate (named B06), ameliorated the cognitive decline, improved the behavior, and restored the relevant hallmarks of two murine models, namely, the senescence-accelerated, mouse-prone 8 (SAMP8) and the familial AD (5xFAD) [7,8]. We initiated the exploration of the chemical opportunities of the highly functionalized, bicyclic α-iminophosphonates by the reduction and addition of nucleophiles to the imine functional group, resulting in structurally new I_2_-IR ligands. The treatment, performed with selected members of the new families, named B06red and BIN05, recovered the transgenic AD *Caenorhabditis elegans* model to the vehicle group (Scheme 1) [9].

More recently, we reported that the treatment with NaOH of bicyclic α-iminophosphonates caused the opening of the imide-containing ring, followed by subsequent decarboxylation. The selected diethyl (2*RS*,3*RS*)-3-((3-chloro-4-fluorophenyl)carbamoyl)-2-phenyl-3,4-dihydro-2*H*-pyrrol-2-yl)phosphonate (named PIP01, **1e**), endowed with optimal ADME-Tox and anti-inflammatory properties, was chosen for treating the murine SAMP8 model and a capsaicin-induced mechanical hypersensitivity model, revealing neuroprotective and analgesic properties (Scheme 1) [10,11].

Encouraged by the synthetic opportunities that bicyclic α-iminophosphonates offer, herein, we report the reduction of the α-iminophosphonate functional group, resulting in (pyrrolidine-2-yl)phosphonates. Azaheterocyclic phosphonates have been pointed out as remarkable entities due to their biological activity [12], and pyrrolidine is the most frequent five-membered, nonaromatic nitrogen heterocycle ring found in US FDA-approved drugs [13,14]. Thus, the reduction in the previously reported (3,4-dihydro-2*H*-pyrrol-2-yl)phosphonates allowed the synthesis of 3-amido-substituted (pyrrolidine-2-yl)phosphonates (Scheme 1, general structure I) [15]. Additionally, the opening of the imide functional group prevented decarboxylation, which resulted in diversely 3,4-substituted (pyrrolidine-2-yl)phosphonates (Scheme 1, general structure II). Therefore, the α-iminophosphonate [16] functional group was transformed into the α-aminophosphonate [17,18], which is recognized as the phosphonic analogue of α-aminoesters and the bioisoster of α-aminoacids [19]. α-Aminophosphonates show interesting potential utility as chiral building blocks for constructing peptidomimetic structures that can be found in antibiotic, anticancer, and antifungal therapeutic agents, and they are endowed with remarkable capabilities as enzyme inhibitors or receptor ligands in pathological conditions related to *α*-amino acid metabolism [20,21,22,23]. The biological properties of α-aminophosphonates are mostly associated with the tetrahedral configuration of the substituents surrounding the phosphorus atom, which mimic the high-energy transition state involved in the peptide bond’s hydrolysis.

The new compounds have a ubiquitous (pyrrolidine-2-yl)phosphonate nucleus (phosphoproline), the phosphonic counterpart of the outstanding heterocycle proline [24,25,26]. The relative stereochemistry of the different substituents of the newly prepared compounds was unequivocally determined after X-ray crystallographic analyses of two representative monocrystals and by the comparison of the ^1^H and ^13^C NMR spectra data with the other members of the family. Considering the localization of the I_2_-IR in the CNS [27] and our interest in addressing neurodegenerative diseases, we assessed the ability of two representative compounds to cross the blood–brain barrier (BBB) by performing a parallel artificial membrane assay (PAMPA), and we determined their pharmacological profile through competition binding studies in human brain tissues with the selective radioligand of the I_2_-IR, [^3^H]2-[(2-benzofuranyl)-2-imidazoline] ([^3^H]-2-BFI)**.**

## 2. Results and Discussion

**Scheme 2:**
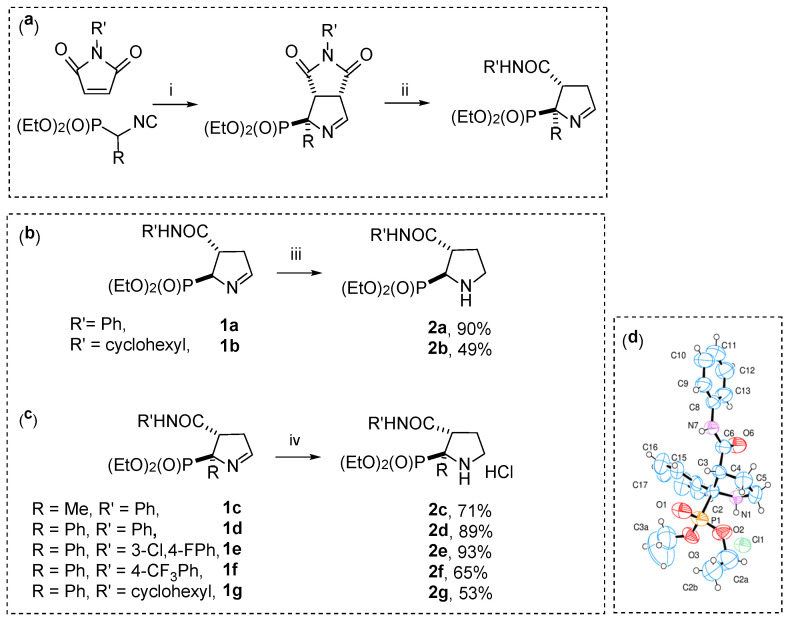
(**a**) General reaction to synthesize starting materials **1a**–**1g**. (**b**) Reduction of (3-phenylcarbamoyl-3,4-dihydro-2*H*-pyrrol-2-yl)phosphonates **1a** and **1b**. (**c**) Reduction of (3-phenylcarbamoyl-3,4-dihydro-2*H*-pyrrol-2-yl)phosphonates **1c**–**1g**. (**d**) X-ray structure of **2d**. Reagents and conditions: i. AgOAc cat., CH_3_CN, rt, overnight (Ref. [1]). ii. NaOH 0.05 M, THF/H_2_O, rt, 2.5 h (Ref. [11]). iii. Pd/C 20%, isopropanol, 300 psi, rt, 8 h. iv. NaBH_3_CN, CH_3_CN/H_2_O/AcOH, rt, 1 h.

**Scheme 3:**
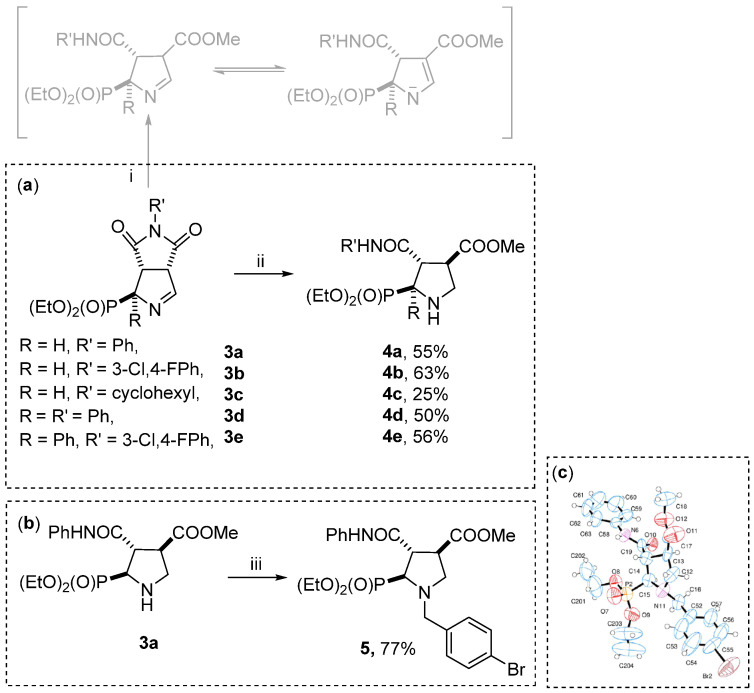
Putative intermediates from the reaction of bicyclic iminophosphonates with Dowex Marathon-A resin in grey. (**a**) Resulting products from the hydrolysis and reduction of bicyclic iminophosphonates **3a**–**3e**. (**b**) Synthesis of **5** containing a heavy atom (Br). (**c**) X-ray of compound **5**. Reagents and conditions: i. Dowex Marathon-A resin, MeOH. ii. Dowex Marathon-A resin, MeOH and H_2_, PtO_2_ (cat), AcOH, rt 24 h or 48 h. iii. NaBH_3_CN, MeOH, AcOH, 4-bromobenzaldehyde, rt, 2 h.

The starting bicyclic iminophosphonates (Scheme 2a and Scheme 3a) were prepared by the diastereoselective [3+2]cycloaddition reaction of diethyl isocyanomethylphosphonate, diethyl α-methylisocyanomethylphosphonate or diethyl α-phenylisocyanomethylphosphonate and an *N*-substituted maleimide, following procedures described by us (Scheme 2a) [1]. Following our previously reported procedure, the opening/descarboxylation of the imide containing ring was performed by the treatment of the bicyclic derivatives with a solution of NaOH 0.05 M in a 2:1 mixture of THF/H_2_O, leading to (3-phenylcarbamoyl-3,4-dihydro-2*H*-pyrrol-2-yl)phosphonate derivatives (Scheme 2a) [11]. First, we carried out the reduction of the imine functional group of the starting compounds **1a** and **1b** with a hydrogen atom in the α-phosphonate position. The hydrogenation of **1a**, at atmospheric pressure using Pd/C 20% as a catalyst in EtOH, EtOAc, THF, CH_2_Cl_2_ or CH_3_CN, was unsuccessful. When more drastic conditions were employed, and the reaction was conducted at pressure (300 psi) in isopropanol, the reduction of the imine group took place to give **2a** in a 90% yield. In a similar manner, the hydrogenation of **1b** gave compound **2b** in a 49% yield (Scheme 2b). These reaction conditions did not allow the transformation of the counterparts, **1c**–**1g**, embodying a substituent in the α-phosphonate position. However, the reduction of **1c**–**1g** with NaBH_3_CN in CH_3_CN after stirring for 2 h at room temperature led to a residue that, after treatment with EtOH^.^HCl (1.25 M), conducted to the hydrochlorides of compounds **2c**–**2g** [28].

In detail, quaternary α-aminophosphonates, with an α-methyl substituent, **2c**, and with an α-phenyl substituent [29], **2d**, **2e**, **2f** and **2g**, were obtained with 71%, 89%, 93%, 65% and 53% yields, respectively (Scheme 2c). The relative configurations of the substituents in the 2- and 3-positions of the pyrrolidine ring were confirmed by X-ray crystallographic analysis of a monocrystal of compound **2d** (see Section 3.2). The 2α-phenylsubstituent and the 3-carboxamido substituents showed a *cis* relationship, as described in the starting materials, confirming that the reduction proceeds without modifying the relative configuration (Scheme 2d).

With the aim of confirming that the stereochemistry in all the new compounds is the same as that unequivocally determined in **2d** (Scheme 2d), we compared the ^1^H and ^13^C-NMR spectra of the different members of the family. In all the compounds **2a**–**2g**, H-3 has a coupling constant of 10.5–13.5 Hz with the phosphorous atom, and a coupling constant of 7.5–8.0 Hz with the H-4. In the ^13^C NMR spectra of the α-monosubstituted derivatives **2a** and **2b**, it can be observed that C-2, 58.0–58.5 ppm, has a coupling constant of 165.0 Hz, and for the α-substituted-2-phosphorylpyrrolidines **2d**, **2e**, **2f**, and **2g**, C-2, 72.0–74.0 ppm, has a coupling constant of 149.5–150.0 Hz. The shield for C-2 and C-5 is in accordance with the proposed relative stereochemistry (Table 1).

**Figure 2:**
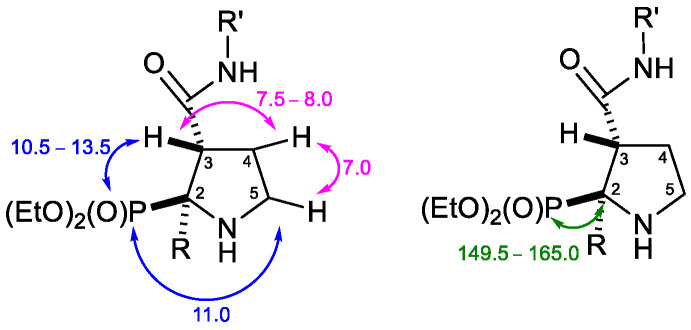
Representative coupling constants (Hz): ^1^H-P (blue), ^1^H-^1^H (pink) and ^13^C-P (green).

Figure 2 summarizes the representative coupling constants in the ^1^H and ^13^C-NMR spectra of **2a**–**2g**, considering the multiplicity due to the phosphorous atom in the phosphonic ester substituent.

Then, we took up the challenge to access diversely substituted (pyrrolidine-2-yl)phosphonates maintaining the substituent in the 4 position. Thus, the opening of the imide group from bicyclic iminophosphonates **3a**–**3e** was induced easily by the filtration of a solution of the product in MeOH through a Dowex MarathonA resin [30], although the desired product was unstable due to the tautomeric imine–enamine equilibrium (Scheme 3, intermediates in grey). Therefore, once the imide was opened, the reduction reaction was performed straightforwardly without the isolation of the intermediates by a hydrogenation reaction using PtO_2_ as a catalyst at rt. In this manner, the compounds **4a**, **4b** and **4c** were synthesized in 55, 63 and 25% yields, respectively, and compounds **4d** and **4e** in 50 and 56% yields, respectively (Scheme 3a). To elucidate the relative stereochemistry of new trisubstituted (**4a**, **4b** and, **4c**) and tetrasubstituted (**4d** and **4e**) pyrrolidine-2-phosphonates, it was necessary to synthetize a derivative containing a heavy atom. Therefore, compound **5** was accessed, after the reductive amination reaction of **4a** and 4-bromobenzaldehyde with NaBH_3_CN in MeOH catalyzed by AcOH at rt for 2 h, in a 77% yield (Scheme 3b). The X-ray crystallographic analysis of **5** revealed that the 2-phosphonate ester substituent and the 2-carboxamido group retain the *trans* configuration of the starting materials. The 4-ester substituent adopts a *trans* relationship with the 3-carboxamido group, probably due to the protonation occurring in the course of the imine–enamine equilibrium, yielding the most favorable face and avoiding 3,4-steric hindrance (Scheme 3c).

The relative configuration in the three stereocenters of the new compounds depicted in Scheme 3b was confirmed by comparison of their ^1^H and ^13^C-NMR spectra with the compound **5**. Apart from the coincident shield of the different peaks, it is remarkable that in the ^13^C NMR spectra of compounds **4a**, **4b** and **4c**, with a hydrogen atom in the α-position, the C-5 appears at 58.0–59.0 ppm with a coupling constant of 162.0–164.0 Hz, and when a phenyl group is present in the α-position, **4d** and **4e**, the C-5 appears at 68.0–69.0 ppm, with a coupling constant of 158.0–159.0 Hz. It can be observed that the carbon atoms near to the phosphonate group show the expected coupling constants. In particular, the C2 appears at 52.1–56.6 ppm with a coupling constant of 10.5–13.5 Hz, with the exception of **4d** and **4e** at 49.0 ppm and with a coupling constant lower than 7.0 Hz, while the C-3 at 44.0–48.0 ppm has a 7.5–8.0 Hz coupling constant in all the new compounds (Table 2).

**Figure 3:**
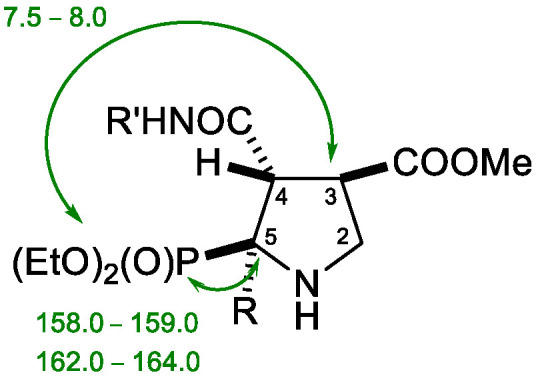
Representative coupling constants (Hz) ^13^C-P (green).

Figure 3 shows representative coupling constants in the ^13^C-NMR spectra of **4a**–**4e**, considering the multiplicity due to the phosphorous atom in the phosphonic ester substituent.

For the sake of comparison with our previous biological studies (Scheme 1), we selected compounds **2e** (Scheme 2c) and **4e** (Scheme 3a) with the substituents R = Ph and R’ = 3-Cl,4-FPh.

First of all, and taking into account the need to have molecules with a good capacity to permeate the BBB for modulating I_2_-IR, the in vitro permeability (Pe) of **2e** and **4e** was determined by using the PAMPA-BBB assay. Both compounds are predicted to achieve high BBB penetration (Pe > 5.030 × 10^−6^ cm s^−1^) with values of Pe = 14.6 ± 0.4 × 10^−6^ cm s^−1^ for **2e** and Pe = 14.35 ± 0.6 × 10^−6^ cm s^−1^ for **4e**.

I_2_-IRs are described as non-adrenergic receptors for imidazolines; therefore, the pharmacological profiles of **2e** and **4e** were evaluated considering both affinity for I_2_-IR and selectivity versus the α_2_-adrenergic receptor (α_2_-AR) [31]. The affinity for I_2_-IR was assessed through competition binding studies against the selective I_2_-IR radioligand [^3^H]2-BFI. The selectivity versus the α_2_-AR was evaluated through competition studies using the selective radioligand [^3^H]RX821002 (2-methoxyidazoxan). The studies were performed in membranes from a post-mortem human frontal cortex, a brain area that shows a significant density of I_2_-IR and α_2_-AR. The inhibition constant (*K*_i_) for each compound was obtained and is expressed as the corresponding p*K*_i_. The selectivity for these two receptors was expressed by the I_2_/α_2_ index, calculated as the antilogarithm of the ratio between p*K*_i_ values for I_2_-IR and p*K*_i_ values for α_2_-AR.

**Figure 4:**
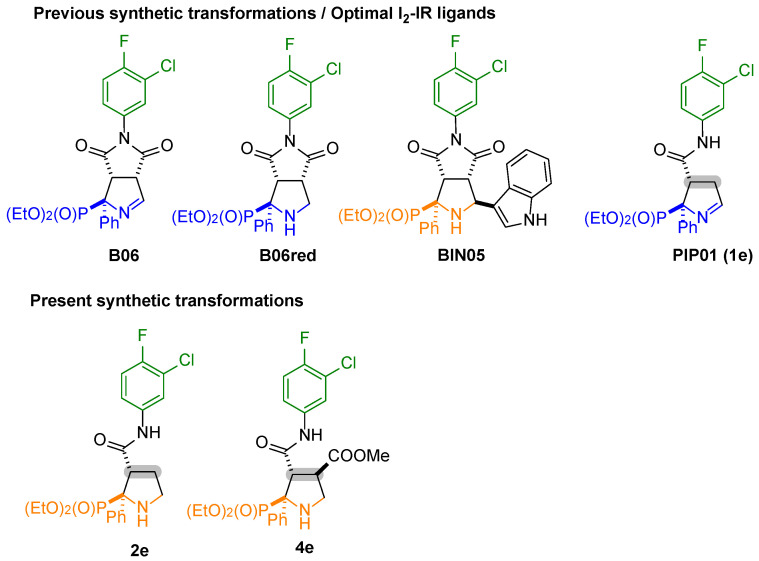
Structure of the four previously reported compounds B06, B06red, BIN05 and PIP01 (**1e**) and the two selected compounds, **2e** and **4e**, evaluated in this paper as I_2_-IR ligands. Highlighted in blue is the α-phenyliminophosphonate moiety, and highlighted in orange is the α-phenylaminophosphonate moiety and in grey the monocycles.

To determine the impacts that the structural modifications in the new compounds selected, **2e** and **4e**, have in the affinity/selectivity data, we considered the values already published by us on the clinical candidates CR4056 and BU99008 [32,33], as well as B06, B06red, BIN05 and PIP01 (**1e**) [1,9,11]. The affinity of these compounds for I_2_-IR is better described with a biphasic curve, except for PIP01 (**1e**). The clinical candidates CR4056 and BU99008 were endowed with a p*K*_iH_ I_2_ = 7.72 ± 0.31 (29% occupancy), p*K*_iL_ I_2_ = 5.45 ± 0.15 (Table 3, entry 1), p*K*_iH_ I_2_ = 6.89 ± 0.21 (51% occupancy) and p*K*_iL_ I_2_ = 3.82 ± 0.30 (Table 3, entry 2), respectively. Next, the members of the new families evaluated that embodied an α-phenyl phosphonate substituent and a 3-Cl, 4-Fphenyl substituent in the nitrogen atom were B06, with p*K*_iH_ I_2_ = 8.61 ± 0.28 (37% occupancy) and p*K*_iL_ I_2_ = 4.29 ± 0.20 (Table 3, entry 3), B06red, which showed an outstanding value fitted into two sites with a p*K*_iH_ of 9.81 ± 0.21 and a 50% occupancy, and without selectivity versus α_2_-AR (Table 3, entry 4), and BIN05, with an affinity that fitted into a two-fold curve with values within the same range (p*K*_iH_ 8.18 and p*K*_iL_ 3.56) of the affinity value for B06, but with a lower occupancy of the high-affine site (21% vs. 37% for B06) and a lack of selectivity upon α_2_-AR (Table 3, entry 5). The opening of the imide-containing ring in compound PIP01 (**1e**) involved a remarkable benefit by increasing the affinity to p*K*_i_ 9.98 ± 0.74, but again without I_2_/*α*_2_ selectivity (Table 3, entry 6). The two selected new compounds **2e** and **4e** share common structural features with the outstanding PIP01 (**1e**) because they are monocyclic phosphonates, substituted in the α-phoshonate position by a phenyl group and by a *p*-fluor atom and a *m*-chlorine atom in the *N*-phenyl group. However, these coincidences did not overcome the deleterious effect on the p*K*_i_ value shown by the reduction of the imino into the amino functional group in **2e**. Note that the reduction of the imino group combined with the presence of a methyl ester group in the C3, **4e**, gave an interesting affinity value (p*K*_i_ 7.99 ± 0.39) and a very good I_2_/*α*_2_ selectivity (58,884) (Figure 4).

## 3. Materials and Methods

### 3.1. Chemistry

#### 3.1.1. General Information

Reagents, solvents and starting products were acquired from commercial sources. The evaporation of solvents was accomplished with a rotary evaporator. The reactions were monitored by thin-layer chromatography (TLC) using silica gel (60 F_254_) plates. Compounds were visualized by UV irradiation and/or spraying with 1% aqueous KMnO_4_. Flash column chromatography was performed on the Biotage^®^ Isolera™ Prime chromatograph equipped with a UV detector with normal Silica Gel Columns (SNAP KP-Sil, silica gel 60 Å 35–70 μm) with the indicated solvent system. Melting points were measured in MFB 59510M Gallenkamp instruments (Loughborough, UK). IR spectra were derived in a spectrophotometer Nicolet Avantar 320 FTR-IR (Elsichrom, Knivsta, Sweden) or in a Spectrum Two FT-IR Spectrometer (PerkinElmer, Springfield, IL, USA), and only noteworthy IR absorptions (cm^−1^) are listed. NMR spectra were recorded in CDCl_3_ or CD_3_OD at 400 MHz (^1^H) and 100.6 MHz (^13^C) in a Varian Mercury 400 MHz or Bruker Avance Neo 400 MHz (Bruker, Billerica, MA, USA), and chemical shifts are reported in *δ* values downfield from TMS or relative to residual solvent (7.26 ppm and 77.0 ppm for CDCl_3_, 3.31 ppm and 49.0 ppm for CD_3_OD) as an internal standard. Data are reported in the following manner: chemical shift, multiplicity, coupling constant (*J*) in hertz (Hz), integrated intensity and assignment (when possible). Multiplicities are reported using the following abbreviations: s, singlet; d, doublet; dd, doublet of doublets; ddd, doublet of doublets of doublets; dddd, doublet of doublets of doublet of doublets; t, triplet; dtd, doublet of triplet of doublets; m, multiplet; c. s., complex signal; br s, broad signal; app, apparent. Assignments and stereochemical determinations are given only when they are derived from definitive two-dimensional NMR experiments (g-HSQC-COSY). The accurate mass analyses were carried out using a LC/MSD-TOF spectrophotometer. The elemental analyses were carried out in a Flash 1112 series Thermofinnigan elemental microanalyzator (A5) to determine C, H, and N.

#### 3.1.2. General Procedure for the Reduction of **1a** and **1b**

Here, 20% Pd/C was added to a solution of **1a** and **1b** in 2-propanol and the mixture was stirred under hydrogen atmosphere pressure (300 psi) at room temperature overnight. Then, the crude mixture was filtered through Celite^®^ and the solvent was evaporated to afford the products **2a** and **2b**.

*Diethyl (2RS,3RS)-3-(phenylcarbamoyl)pyrrolidine-2-phosphonate* (**2a**). Following the general procedure, 20% Pd/C (22 mg), **1a** (108 mg, 0.33 mmol) and 2-propanol (15 mL) gave **2a** (97 mg, 90%) as a yellowish oil. IR (NaCl) 3262, 2980, 2931, 1685, 1551, 1233, 1028, 968, 791, 756, 693 cm^−1^. ^1^H NMR (400 MHz, CDCl_3_) *δ* 1.31 (t, *J* = 7.0 Hz, 3H, CH_2_C*H*_3_), 1.38 (t, *J* = 7.0 Hz, 3H, CH_2_C*H*_3_), 2.06 (m, 1H, H-4), 2.19–2.29 (c.s., 2H, H-4 and NH), 2.99–3.12 (c.s., 2H, H-5), 3.26 (ddd, *J* = 24.0, 16.0, 8.0 Hz, 1H, H-3), 3.63 (t, *J* = 7.5 Hz, 1H, H-2) 4.10–4.30 (m, 4H, C*H*_2_CH_3_), 7.06 (t, *J* = 7.5 Hz, 1H, ArH), 7.27 (m, 2H, ArH), 7.62 (m, 2H, ArH), 9.44 (br s, 1H, NH). ^13^C NMR (100.6 MHz) *δ* 16.4 (d, *J* = 5.5 Hz, CH_2_*C*H_3_), 16.5 (d, *J* = 5.5 Hz, CH_2_*C*H_3_), 31.2 (d, *J* = 7.0 Hz, C-4), 47.5 (d, *J* = 2.0 Hz C-3), 48.4 (d, *J* = 9.0 Hz C-5), 57.8 (d, *J* = 165.0 Hz, C-2), 62.8 (d, *J* = 7.0 Hz, *C*H_2_CH_3_), 63.1 (d, *J* = 7.0 Hz, *C*H_2_CH_3_), 119.5 (2CHAr), 123.7 (CHAr), 128.8 (2CHAr), 138.7 (C-*ipso*), 170.9 (d, *J* = 5.0 Hz, CO). MS-EI *m*/*z* 326 M^+^ (16), 205 (9), 189 (100), 150 (12), 93 (29), 70 (50). HRMS C_15_H_24_N_2_O_4_P [M + H]^+^ 327.1466; found, 327.1468.

*Diethyl (2RS,3RS)-3-(cyclohexylcarbamoyl)pyrrolidine-2-phosphonate* (**2b**). Following the general procedure, 20% Pd/C (22 mg), **1b** (108 mg, 0.33 mmol) and 2-propanol (15 mL) gave **2b** (53 mg, 49%) as a white solid after column chromatography (CH_2_Cl_2_/MeOH 95:5). IR (NaCl) 3293, 2931, 2855, 1649, 1547, 1234, 1056, 966, 891, 790 cm^−1^. ^1^H NMR (400 MHz, CDCl_3_) *δ* 1.13–1.23 (m, 3H, CH_2_cycl), 1.30–1.41 (m, 2H, CH_2_cycl), 1.34 (t, *J* = 7.0, Hz, 3H, CH_2_C*H*_3_), 1.35 (t, *J* = 7.0 Hz, 3H, CH_2_C*H*_3_), 1.59 (m, 1H, CH_2_cycl), 1.69–1.73 (m, 2H, CH_2_cycl), 1.86–1.90 (m, 2H, CH_2_cycl), 1.97 (m, 1H, H-4), 2.13–2.22 (c.s., 2H, H-4 and NH), 2.91–3.07 (c.s., 3H, CHcycl and H-5), 3.46 (t, *J* = 7.5 Hz, 1H, H-2), 3.75 (m, 1H, H-3), 4.13–4.25 (m, 4H, C*H*_2_CH_3_), 6.56 (d, *J* = 7.5 Hz, 1H, NH). ^13^C NMR (100.6 MHz) *δ* 16.4 (d, *J* = 6.0 Hz, CH_2_*C*H_3_), 16.5 (d, *J* = 6.0 Hz, CH_2_*C*H_3_), 24.7 (2CH_2_cycl), 25.5 (CH_2_cycl) 30.9 (d, *J* = 6.5 Hz, C-4), 32.8 (CH_2_cycl), 32.9 (CH_2_cycl), 46.6 (CHcycl), 48.2 (C-5), 48.4 (d, *J* = 9.5 Hz, C-3), 58.5 (d, *J* = 165.0 Hz, C-2), 62.6 (d, *J* = 7.0 Hz, *C*H_2_CH_3_), 62.8 (d, *J* = 7.0 Hz, *C*H_2_CH_3_), 171.7 (d, *J* = 5.5 Hz, CO). MS-EI *m*/*z* 332 M^+^ (11), 195 (100), 178 (15), 150 (15), 111 (7), 96 (10), 83 (13), 70 (62). HRMS C_15_H_30_N_2_O_4_P [M + H]^+^ 333.1944; found, 333.1938.

#### 3.1.3. General Procedure for the Reduction of **1c**, **1d**, **1e**, **1f** and **1g**

To a solution of CH_3_CN and H_2_O, 1-pyrroline **1c, 1d, 1e, 1f** and **1g** (0.1 mmol) was added and the mixture was stirred for 10 min. Then, NaBH_3_CN (0.2 mmol) and AcOH were added, and the mixture was stirred for 1h at rt. The solvent was evaporated, EtOAc was added to the residue and the resulting solution was washed with a saturated solution of NaHCO_3_. The organic phase was dried and concentrated in vacuo. The residue was suspended in CH_2_Cl_2_, a solution of HCl·EtOH 1.25 M was added, and the resulting precipitated solid was collected by filtration to yield **2c**, **2d**, **2e**, **2f** and **2g**.

*Diethyl (2RS,3RS)-2-methyl-3-(phenylcarbamoyl)pyrrolidine-2-phosphonate hydrochloride* (**2c**). Following the general procedure, **1c** (15 mg, 0.04 mmol), CH_3_CN (0.4 mL), H_2_O (20 μL), NaBH_3_CN (6 mg, 0.09 mmol) and AcOH (20 μL) gave **2c** (11 mg, 71%) as a colorless oil. IR (NaCl) 2981, 1686, 1600, 1547, 1443, 1389, 1311, 1249, 1020, 756, 693 cm^−1^. ^1^H NMR (400 MHz, CD_3_OD) *δ* 1.41 (t, *J* = 7.0 Hz, 3H, CH_2_C*H*_3_), 1.43 (t, *J* = 7.0 Hz, 3H, CH_2_C*H*_3_), 1.68 (d, *J* = 15.2 Hz, 3H, CH_3_), 2.43–2.53 (c.s., 2H, H-4), 3.48 (ddd, *J* = 11.2, 8.4, 6.8 Hz, 1H, H-5), 3.58 (ddd, *J* = 13.6, 7.6, 6.0 Hz, 1H, H-3), 3.69 (ddd, *J* = 11.2, 8.4, 6.8 Hz, 1H, H-5), 4.29–4.39 (m, 4H, C*H*_2_CH_3_), 7.13 (m, 1H, ArH), 7.30–7.35 (m, 2H, ArH), 7.56–7.61 (m, 2H, ArH). ^13^C NMR (100.6 MHz) *δ* 15.2 (d, *J* = 5.0 Hz, CH_2_*C*H_3_), 15.3 (d, *J* = 5.0 Hz, CH_2_*C*H_3_), 15.5 (*C*H_3_), 27.8 (d, *J* = 4.0 Hz, C-4), 45.2 (d, *J* = 3.0 Hz, C-5), 48.8 (C-3), 64.4 (d, *J* = 159.0 Hz, C-2), 64.7 (d, *J* = 7.5 Hz, *C*H_2_CH_3_), 65.1 (d, *J* = 7.5 Hz, *C*H_2_CH_3_), 119.9 (2CHAr), 124.4 (CHAr), 128.5 (2CHAr), 137.7 (C-*ipso*), 168.1 (d, *J* = 9.0 Hz, CO). HRMS C_16_H_26_N_2_O_4_P [M + H]^+^ 341.1625; found, 341.1625.

*Diethyl (2RS,3SR)-2-phenyl-3-phenylcarbamoylpyrrolidine-2-phosphonate hydrochloride* (**2d**). Following the general procedure, **1d** (100 mg, 0.25 mmol), CH_3_CN (2.5 mL), H_2_O (125 μL), NaBH_3_CN (31 mg, 0.5 mmol) and AcOH (125 μL) gave **2d** (98 mg, 89%) as a white solid. M.p. 132–134 °C. IR (NaCl) 3434, 3049, 2979, 1681, 1552, 1254, 1056, 977, 795, 759, 698, 573, 543 cm^−1^. ^1^H NMR (400 MHz, CD_3_OD) *δ* 1.14 (t, *J* = 7.0 Hz, 3H, CH_2_C*H*_3_), 1.34 (t, *J* = 7.0 Hz, 3H, CH_2_C*H*_3_), 2.42 (m, 1H, H-4), 2.83 (m, 1H, H-4), 3.53 (m, 1H, C*H*_2_CH_3_), 3.81–3.89 (c.s., 3H, H-5 and C*H*_2_CH_3_), 4.09–4.19 (m, 2H, C*H*_2_CH_3_), 4.32 (dd, *J* = 10.8, 7.6 Hz, 1H, H-3) 7.04 (dt, *J* = 7.2, 1.2 Hz, 1H, ArH), 7.18–7.22 (m, 2H, ArH), 7.30–7.33 (m, 2H, ArH), 7.38–7.50 (m, 6H, ArH and NH). ^13^C NMR (100.6 MHz) *δ* 15.0 (CH_2_*C*H_3_), 15.1 (CH_2_*C*H_3_), 28.3 (C-4), 45.8 (C-5), 49.9 (C-3), 64.7 (d, *J* = 8.0 Hz, *C*H_2_CH_3_), 65.7 (d, *J* = 7.5 Hz, *C*H_2_CH_3_), 72.8 (d, *J* = 149.5 Hz, C-2), 120.0 (2CHAr), 124.3 (CHAr), 126.6 (d, *J* = 4.0 Hz, 2CHAr), 128.3 (2CHAr), 128.6 (d, *J* = 2.5 Hz, 2CHAr), 128.9 (d, *J* = 3.0 Hz, CHAr) 132.6 (d, *J* = 4.0 Hz, C-*ipso*), 137.4 (C-*ipso*), 170.3 (d, *J* = 17.5 Hz, CO). MS-EI *m*/*z* 402 M^+^ (0.6), 264 (100), 172 (77), 144 (86), 117 (53), 93 (76), 69 (87), 41 (33). Anal. Cald. for C_21_H_28_ClN_2_O_4_P: C, 57.47; H, 6.43; N, 6.38. Found: C, 57.08; H, 6.44; N, 6.40%.

*Diethyl (2RS,3SR)-3-(3-chloro-4-fluorophenyl)carbamoyl)-2-phenylpyrrolidine-2-phosphonate hydrochloride* (**2e**). Following the general procedure, **1e** (56 mg, 0.12 mmol), CH_3_CN (1.2 mL), H_2_O (60 μL), NaBH_3_CN (15 mg, 0.25 mmol) and AcOH (60 μL) gave **2e** (31 mg, 53%) as a white solid. M.p. 115–116 °C. IR (NaCl) 3460, 2978, 2929, 1684, 1500, 1230, 1052, 1021, 978, 750, 700, 565, 525 cm^−1^. ^1^H NMR (400 MHz, CD_3_OD) *δ* 1.14 (t, *J* = 7.0 Hz, 3H, CH_2_C*H*_3_), 1.34 (t, *J* = 7.0 Hz, 3H, CH_2_C*H*_3_), 2.43 (m, 1H, H-4), 2.83 (m, 1H, H-4), 3.54 (m, 1H, C*H*_2_CH_3_), 3.79–3.91 (c.s., 3H, H-5 and C*H*_2_CH_3_), 4.09–4.20 (m, 2H, C*H*_2_CH_3_), 4.30 (dd, *J* = 10.4, 7.6 Hz, 1H, H-3) 7.11 (t, *J* = 10.0 Hz, 1H, ArH), 7.24 (m, 1H, ArH), 7.40–7.46 (m, 5H, ArH), 7.58 (dd, *J* = 6.5, 2.5 Hz, 1H, ArH). ^13^C NMR (100.6 MHz) *δ* 15.0 (CH_2_*C*H_3_), 15.1 (CH_2_*C*H_3_), 28.2 (C-4), 45.8 (C-5), 50.0 (C-3), 64.8 (d, *J* = 8.0 Hz, *C*H_2_CH_3_), 65.8 (d, *J* = 7.5 Hz, *C*H_2_CH_3_), 72.7 (d, *J* = 150.0 Hz, C-2), 116.1 (d, *J* = 22.5 Hz, CHAr), 119.8 (d, *J* = 7.0 Hz, CHAr), 121.7 (CHAr), 121.8 (d*, J* = 2.0 Hz, C-*ipso*), 126.6 (d, *J* = 4.0 Hz, 2CHAr), 128.6 (CHAr), 128.7 (CHAr), 129.0 (d, *J* = 3.0 Hz, CHAr), 132.5 (d, *J* = 3.5 Hz, C-*ipso*), 134.5 (d, *J* = 3.0 Hz, C-*ipso*), 154.6 (d, *J* = 245.5 Hz, C-*ipso*), 170.4 (d, *J* = 17.5 Hz, CO). MS-EI *m*/*z* 454 M^+^ (0.5), 358 (12), 317 (100), 213 (36), 172 (60), 144 (66), 117 (47), 83 (29), 69 (77). HRMS C_21_H_25_ClFN_2_O_4_P [M + H]^+^ 454.1224; found, 454.1226.

*Diethyl ((2RS,3SR)-2-phenyl-3-((4-(trifluoromethyl)phenyl)carbamoyl)pyrrolidin-2-yl)phosphonate* (**2f**). Following the general procedure, **1f** (15 mg, 0.04 mmol), CH_3_CN (0.4 mL), H_2_O (20 μL), NaBH_3_CN (6 mg, 0.09 mmol) and AcOH (20 μL) gave **2f** (11 mg, 71%) as a colorless oil. ^1^H NMR (400 MHz, CD_3_OD) *δ* 1.14 (t, *J* = 7.0 Hz, 3H, CH_2_C*H*_3_), 1.37 (t, *J* = 7.0 Hz, 3H, CH_2_C*H*_3_), 2.49 (m, 1H, H-4), 2.84 (m, 1H, H-4), 3.38–3.62 (m, 1H, C*H*_2_CH_3_), 3.74–3.98 (c.s., 3H, H-5 and C*H*_2_CH_3_), 4.05–4.24 (m, 2H, C*H*_2_CH_3_), 4.28 (dd, *J* = 10.5, 7.5 Hz, 1H, H-3), 7.30–7.39 (m, 1H, ArH), 7.40–7.45 (m, 2H, 2ArH), 7.45–7.50 (m, 4H, ArH), 7.50–7.57 (m, 1H, ArH), 7.76 (m, 1H, ArH). ^13^C NMR (100.6 MHz, CD_3_OD) *δ* 16.4 (CH_2_*C*H_3_), 16.5 (CH_2_*C*H_3_), 29.7 (C-4), 47.2 (C-5), 51.6 (C-3), 66.2 (d, *J* = 8.5 Hz, *C*H_2_CH_3_), 67.2 (d, *J* = 7.5 Hz, *C*H_2_CH_3_), 74.2 (d, *J* = 149.5 Hz, C-2), 117.6 (CHAr), 122.0 (CHAr), 124.3 (CHAr), 127.9 (2CHAr), 128.0 (q, *J* = 272.5 Hz, *C*F_3_),130.1 (2CHAr), 130.5 (C-*ipso*), 130.8 (2CHAr), 132.1 (q, *J* = 32.5 Hz, *C*CF_3_), 139.8 (C-*ipso*), 172.1 (d, *J* = 18.0 Hz, CO).

*Diethyl (2RS,3SR)-3-cyclohexylcarbamoyl-2-phenylpyrrolidine-2-phosphonate hydrochlorid*e (**2g**). Following the general procedure, **1g** (83 mg, 0.20 mmol), CH_3_CN (2.0 mL), H_2_O (100 μL), NaBH_3_CN (25 mg, 0.40 mmol) and AcOH (100 μL) gave **2g** (83 mg, 93%) as a white solid. M.p. 172 °C. IR (NaCl) 3425, 2931, 2855, 1659, 1552, 1242, 1052, 982, 751, 699, 668, 552 cm^−1^. ^1^H NMR (400 MHz, CD_3_OD) *δ* 0.98 (m, 1H, CH_2_cycl), 1.26 (t, *J* = 7.0, Hz, 3H, CH_2_C*H*_3_), 1.17–1.36 (m, 5H, CH_2_cycl), 1.32 (t, *J* = 7.0 Hz, 3H, CH_2_C*H*_3_), 1.54–1.61 (m, 2H, CH_2_cycl), 1.70–1.74 (m, 2H, CH_2_cycl), 2.25 (m, 1H, H-4), 2.73 (m, 1H, H-4), 3.35 (m, 1H, CHcycl), 3.52 (m, 1H, C*H*_2_CH_3_), 3.77–3.88 (c.s., 3H, C*H*_2_CH_3_ and H-5), 4.06–4.17 (c.s., 3H, C*H*_2_CH_3_ and H-3), 7.40–7.49 (m, 5H, ArH). ^13^C NMR (100.6 MHz) *δ* 15.0 (CH_2_*C*H_3_), 15.1 (CH_2_*C*H_3_), 24.4 (CH_2_cycl), 24.5 (CH_2_cycl), 25.1 (CH_2_cycl) 28.1 (C-4), 31.9 (CH_2_cycl), 32.0 (CH_2_cycl), 45.9 (C-5), 48.0 (CHcycl), 49.1 (C-3), 64.7 (d, *J* = 8.0 Hz, *C*H_2_CH_3_), 65.7 (d, *J* = 7.5 Hz, *C*H_2_CH_3_), 72.5 (d, *J* = 149.5 Hz, C-2), 126.8 (d, *J* = 4.0 Hz, 2CHAr), 128.4 (d, *J* = 2.5 Hz, 2CHAr), 128.8 (d, *J* = 3.0 Hz, CHAr), 132.6 (d, *J* = 3.5 Hz, C-*ipso*), 170.8 (d, *J* = 17.0 Hz, CO). MS-EI *m*/*z*; 408 M^+^ (1), 270 (76), 172 (10), 144 (100), 117 (19), 86 (17), 69 (19). HRMS C_21_H_34_N_2_O_4_P [M + H]^+^ 409.2261; found, 409.2251.

#### 3.1.4. General Procedure for the Treatment with Resin and Hydrogenation of **3a**, **3b**, **3c**, **3d**, and **3e**

Bicyclic iminophosphonates **3a**, **3b**, **3c**, **3d** and **3e** were passed through a Dowex Marathon-A^®^ resin with MeOH (50 mL). Then, the solvent was evaporated, and the resulting oil was dissolved in AcOH. PtO_2_ was added (10% for compound **4a**, and 20% for compounds **4b**, **4c**, **4d** and **4e**) and the suspension was vigorously stirred under hydrogen atmosphere at rt for 1 day (compound **4a**) and for 2 days (compounds **4b**, **4c**, **4d** and **4e**). The catalyst was removed by filtration with Celite^®^ and the solvent was evaporated to afford a residue, which was purified by column chromatography.

*Methyl (3RS,4SR,5SR)-5-(diethoxyphosphoryl)-4-(phenylcarbamoyl)pyrrolidine-3-carboxylate* (**4a**). Following the general procedure, **3a** (200 mg, 0.57 mmol), MeOH (50.0 mL), AcOH (3.0 mL), PtO_2_ (13 mg, 0.06 mmol) gave **4a** (121 mg, 55%) as a colorless oil, after flash column chromatography (AcOEt/Cyclohexane, SNAP 10 g). IR (NaCl) 3299, 2981, 1734, 1685, 1444, 1229, 1027, 971, 794, 757, 693 cm^−1^. ^1^H NMR (400 MHz, CDCl_3_) *δ* 1.34 (t, *J* = 7.0 Hz, 3H, CH_2_C*H*_3_), 1.40 (t, *J* = 7.0 Hz, 3H, CH_2_C*H*_3_), 2.18 (brs, 1H, NH), 3.22–3.33 (c.s., 2H, H-2), 2.53–3.67 (c.s., 3H, H-3, H-4 and H-5), 3.73 (s, 3H, OCH_3_), 4.18–4.32 (m, 4H,C*H*_2_CH_3_), 7.08 (t, *J* = 7.5 Hz, 1H, ArH), 7.28–7.32 (m, 2H, ArH), 7.58 (d, *J* = 7.6 Hz, 2H, ArH), 9.37 (s, 1H, NH),^13^C NMR (100.6 MHz) *δ* 16.3 (d, *J* = 6.0 Hz, CH_2_*C*H_3_), 16.5 (d, *J* = 5.5 Hz, CH_2_*C*H_3_), 48.2 (d, *J* = 7.5 Hz, C-3), 51.2 (d, *J* = 2.5 Hz, C-4), 52.1 (d, *J* = 11.5 Hz, C-2), 52.3 (OCH_3_), 58.9 (d, *J* = 163.0 Hz, C-5), 63.0 (d, *J* = 7.0 Hz, *C*H_2_CH_3_), 63.5 (d, *J* = 7.0 Hz, *C*H_2_CH_3_), 119.5 (2CHAr), 123.9 (CHAr), 128.9 (2CHAr), 138.4 (C-*ipso*), 168.7 (d, *J* = 3.5 Hz, CO), 173.8 (CO). MS-EI *m*/*z* 384 M^+^ (27), 247 (100), 204 (33), 187 (24), 154 (23), 126 (31), 93 (29), 68 (53). HRMS C_17_H_26_N_2_O_6_P [M + H]^+^ 385.1539; found, 385.1523.

*Methyl (3RS,4SR,5SR)-4-((3-chloro-4-fluorophenyl)carbamoyl)-5-(diethoxyphosphoryl)pyrrolidine-3-carboxylate* (**4b**). Following the general procedure, **3b** (198 mg, 0.48 mmol), MeOH (50.0 mL), AcOH (3.0 mL), PtO_2_ (11 mg, 0.05 mmol) gave **4b** (130 mg, 63%) as a colorless oil, after flash column chromatography (AcOEt/Hexane). IR (NaCl) 3274, 2985, 1719, 1685, 1501, 1395, 1227, 1024, 975, 819, 758 cm^−1^. ^1^H NMR (400 MHz, CDCl_3_) *δ* 1.34 (t, *J* = 7.0 Hz, 3H, CH_2_C*H*_3_), 1.41 (t, *J* = 7.0 Hz, 3H, CH_2_C*H*_3_), 2.62 (br s, 1H, NH), 3.25 (m, 1H, H-2), 3.31 (m, 1H, H-2), 3.48–3.65 (c.s., 3H, H-3, H-4 and H-5), 3.73 (s, 3H, OCH_3_), 4.17–4.24 (m, 2H, C*H*_2_CH_3_), 4.28–4.33 (m, 2H, C*H*_2_CH_3_), 7.06 (t, *J* = 7.0 Hz, 1H, ArH), 7.43 (m, 1H, ArH), 7.79 (dd, *J* = 7.0, 3.0 Hz, 1H, ArH), 9.75 (d, *J* = 10.8 Hz, 1H, NH). ^13^C NMR (100.6 MHz) *δ* 16.3 (d, *J* = 6.0 Hz, CH_2_*C*H_3_), 16.5 (d, *J* = 5.5 Hz, CH_2_*C*H_3_), 47.6 (d, *J* = 8.0 Hz, C-3), 51.1 (d, *J* = 2.2 Hz, C-4), 52.1 (d, *J* = 12.0 Hz, C-2), 52.4 (OCH_3_), 58.8 (d, *J* = 162.0 Hz, C-5), 63.3 (d, *J* = 7.0 Hz, *C*H_2_CH_3_), 63.8 (d, *J* = 7.0 Hz, *C*H_2_CH_3_), 116.4 (d, *J* = 22.0 Hz, CHAr), 119.0 (d, *J* = 6.5 Hz, CHAr), 121.0 (d, *J* = 18.0 Hz, CHAr), 121.5 (CAr), 135.1 (d, *J* = 3.5 Hz, C-*ipso*), 154.5 (d, *J* = 245.5 Hz, C-*ipso*), 168.6 (d, *J* = 2.5 Hz, CO), 173.8 (CO). MS-EI *m*/*z* 436 M^+^ (23), 405 (29), 299 (100), 239 (30), 204 (37), 154 (40), 126 (56), 94 (22), 68 (82). HRMS C_17_H_24_ClFN_2_O_6_P [M + H]^+^ 437.1059; found, 437.1039.

*Methyl (3RS,4SR,5SR)-4-(cyclohexylcarbamoyl)-5-(diethoxyphosphoryl)pyrrolidine-3-carboxylate* (**4c**). Following the general procedure, **3c** (129 mg, 0.36 mmol), MeOH (50.0 mL), AcOH (3.0 mL), PtO_2_ (8 mg, 0.04 mmol) gave **4c** (35 mg, 25%) as a colorless oil, after column chromatography (AcOEt/MeOH 99:1 to AcOEt/MeOH 98:2). IR (NaCl) 3286, 2931, 1735, 1653, 1449, 1235, 1028, 969, 753 cm^−1^. ^1^H NMR (400 MHz, CDCl_3_) *δ* 1.16–1.25 (m, 3H, CH_2_cycl), 1.31–1.38 (m, 2H, CH_2_cycl), 1.35 (t, *J* = 7.0, Hz, 3H, CH_2_C*H*_3_), 1.36 (t, *J* = 7.0 Hz, 3H, CH_2_C*H*_3_), 1.58 (m, 1H, CH_2_cycl), 1.68–1.73 (m, 2H, CH_2_cycl), 1.85–1.89 (m, 2H, CH_2_cycl), 2.19 (br s, 1H, NH), 3.19–3.28 (c.s., 2H, H-2), 3.34 (ddd, *J* = 19.2, 8.0, 6.8 Hz, H-4), 3.45 (d, *J* = 8.8 Hz, 1H, H-5), 3.51 (m, 1H, CHcycl), 3.74 (m 1H, H-3), 3.70 (OCH_3_), 4.14–4.26 (m, 4H, C*H*_2_CH_3_), 6.85 (d, *J* = 8.0 Hz, 1H, NH). ^13^C NMR (100.6 MHz) *δ* 16.4 (d, *J* = 6.0 Hz, CH_2_*C*H_3_), 16.5 (d, *J* = 5.5 Hz, CH_2_*C*H_3_), 24.6 (2CH_2_cycl), 25.5 (CH_2_cycl), 32.7 (CH_2_cycl), 32.8 (CH_2_cycl), 48.4 (CHcycl), 48.5 (d, *J* = 7.0 Hz, C-3), 50.0 (d, *J* = 2.0 Hz, C-4), 51.9 (d, *J* = 10.5 Hz, C-2), 52.1 (OCH_3_), 59.0 (d, *J* = 164.0 Hz, C-5), 62.8 (d, *J* = 7.0 Hz, *C*H_2_CH_3_), 63.2 (d, *J* = 7.0 Hz, *C*H_2_CH_3_), 169.6 (d, *J* = 3.5 Hz, CO), 173.8 (CO). MS-EI *m*/*z* 390 M^+^ (15), 253 (100), 236 (15), 204 (12), 154 (18), 128 (18), 83 (12), 68 (40). HRMS C_17_H_32_N_2_O_6_P [M + H]^+^ 391.1997; found, 391.1992.

*Methyl (3RS,4SR,5RS)-5-(diethoxyphosphoryl)-5-phenyl-4-(phenylcarbamoyl)pyrrolidine-3-carboxylate* (**4d**). Following the general procedure, **3d** (103 mg, 0.24 mmol), MeOH (50.0 mL), AcOH (3.0 mL), PtO_2_ (11 mg, 0.05 mmol) gave **4d** (55 mg, 50%) as a colorless oil, after column chromatography (AcOEt/Hexane 6:4). IR (NaCl) 3275, 3058, 2982, 1733, 1694, 1552, 1444, 1220, 1050, 968, 793, 754, 695, 571 cm^−1^; ^1^H NMR (400 MHz, CDCl_3_) *δ* 1.08 (t, *J* = 7.0 Hz, 3H, CH_2_C*H*_3_), 1.45 (t, *J* = 7.0 Hz, 3H, CH_2_C*H*_3_), 2.65 (br s, 1H, NH), 3.31 (t, *J* = 9.5 Hz, 1H, H-2), 3.62–3.81 (c.s., 3H, H-2, H-3 and C*H*_2_CH_3_), 3.72 (s, 3H, OCH_3_), 3.95–4.05 (c.s., 2H, H-4 and C*H*_2_CH_3_), 4.29–4.38 (m, 2H, C*H*_2_CH_3_), 7.03 (m, 1H, ArH), 7.19–7.25 (m, 5H, ArH), 7.35–7.37 (m, 2H, ArH), 7.67–7.70 (m, 2H, ArH), 9.47 (br s, 1H, NH); ^13^C NMR (100.6 MHz) *δ* 16.1 (d, *J* = 5.5 Hz, CH_2_*C*H_3_), 16.5 (d, *J* = 5.5 Hz, CH_2_*C*H_3_), 46.2 (d, *J* = 7.5 Hz, C-3), 49.2 (d, *J* = 7.0 Hz, C-2), 52.3 (OCH_3_), 57.2 (d, *J* = 4.0 Hz, C-4), 63.7 (d, *J* = 7.5 Hz, *C*H_2_CH_3_), 64.2 (d, *J* = 7.5 Hz, *C*H_2_CH_3_), 68.7 (d, *J* = 158.0 Hz, C-5), 119.8 (2CHAr), 123.8 (CHAr), 127.2 (2CHAr), 128.1 (d, *J* = 2.0 Hz, CHAr), 128.3 (d, *J* = 1.5 Hz, 2CHAr), 128.6 (2CHAr), 136.9 (d, *J* = 5.0 Hz, C-*ipso*), 138.0 (C-*ipso*), 166.4 (d, *J* = 3.0 Hz, CO), 173.4 (CO); MS-EI *m*/*z* 460 M^+^ (0.7), 323 (100), 230 (17), 202 (30), 160 (45), 144 (71), 127 (44), 93 (36); HRMS C_23_H_30_N_2_O_6_P [M + H]^+^ 461.1847; found, 461.1836.

*Methyl (3RS,4SR,5RS)-4-((3-chloro-4-fluorophenyl)carbamoyl)-5-(diethoxyphosphoryl)-5-phenylpyrrolidine-3-carboxylate* (**4e**). Following the general procedure, **3e** (100 mg, 0.21 mmol), MeOH (50.0 mL), AcOH (3.0 mL), PtO_2_ (10 mg, 0.04 mmol) gave **4e** (60 mg, 56%) as a yellowish oil, after flash column chromatography with Biotage (AcOEt/Hexane, SNAP 10 g). IR (NaCl) 3260, 3059, 2983, 1736, 1692, 1500, 1223, 1051, 969, 814, 753, 701 cm^−1^; ^1^H NMR (400 MHz, CDCl_3_) *δ* 1.10 (t, *J* = 7.0 Hz, 3H, CH_2_C*H*_3_), 1.46 (t, *J* = 7.0 Hz, 3H, CH_2_C*H*_3_), 2.63 (br s, 1H, NH), 3.29 (m, 1H, H-2), 3.64–3.70 (c.s., 2H, H-2 and H-3), 3.72 (s, 3H, OCH_3_), 3.80 (m, 1H, C*H*_2_CH_3_), 3.94–4-07 (c.s., 2H, H-4 and C*H*_2_CH_3_), 4.30–4.40 (m, 2H, C*H*_2_CH_3_), 6.95 (t, *J* = 9.0 Hz, 1H, ArH), 7.17–7.27 (m, 4H, ArH), 7.53 (dd, *J* = 7.0, 3.0 Hz, 1H, ArH), 7.64–7.67 (m, 2H, ArH), 9.74 (br s, 1H, NH); ^13^C NMR (100.6 MHz) *δ* 16.1 (d, *J* = 5.5 Hz, CH_2_*C*H_3_), 16.5 (d, *J* = 5.5 Hz, CH_2_*C*H_3_), 45.7 (d, *J* = 7.5 Hz, C-3), 49.3 (d, *J* = 7.0 Hz, C-2), 52.3 (OCH_3_), 57.2 (d, *J* = 3.5 Hz, C-4), 63.9 (d, *J* = 7.5 Hz, *C*H_2_CH_3_), 64.3 (d, *J* = 7.5 Hz, *C*H_2_CH_3_), 68.4 (d, *J* = 159.0 Hz, C-5), 116.3 (d, *J* = 22.0 Hz, CHAr), 119.3 (d. *J* = 6.5 Hz, 2CHAr), 120.7 (d, *J* = 18.5 Hz, C-*ipso*), 121.8 (CHAr), 127.2 (d, *J* = 6.0 Hz, 2CHAr), 128.2 (CHAr), 128.3 (CHAr), 134.7 (d, *J* = 3.0 Hz, C-*ipso*), 137.0 (d, *J* = 6.0 Hz, C-*ipso*), 154.5 (d, *J* = 245.0 Hz, C-*ipso*), 166.4 (d, *J* = 1.5 Hz, CO), 173.2 (CO); MS-EI *m*/*z* 512 M^+^ (0.4), 375 (85), 315 (14), 230 (21), 202 (48), 170 (38), 144 (100), 127 (63), 83 (23); HRMS C_23_H_28_ClFN_2_O_6_P [M + H]^+^ 513.1365; found, 513.1352.

#### 3.1.5. Reductive Amination Reaction

*Methyl (3RS,4SR,5SR)-1-(4-bromobenzyl)-5-(diethoxyphosphoryl)-4-(phenylcarbamoyl)pyrrolidine-3-carboxylate* (**5**). To a solution of 4a (20 mg, 0.05 mmol) in dry MeOH (0.5 mL) were added NaBH_3_CN (7 mg, 0.11 mmol), AcOH (17 μL) and 4-bromobenzaldehyde (14 mg, 0.07 mmol) and the mixture was stirred at room temperature for 2 h. Additional portions of NaBH_3_CN (4 mg, 0.06 mmol) and 4-bromobenzaldehyde (7 mg, 0.03 mmol) were added and the mixture was stirred at room temperature overnight. The solvent was evaporated, EtOAc was added to the residue and the resulting solution was washed with a saturated solution of NaHCO_3_. The organic phase was dried over Na_2_SO_4_, filtered and concentrated to afford 5 (22 mg, 77%), as colorless crystals, after column chromatography (AcOEt/Hexane 1:1). IR (NaCl) 3305, 2925, 1738, 1688, 1601, 1552, 1444, 1251, 1024, 974, 756, 693 cm^−1^; ^1^H NMR (400 MHz, CDCl_3_) δ 1.32 (t, *J* = 7.0 Hz, 3H, CH_2_CH_3_), 1.37 (t, *J* = 7.0 Hz, 3H, CH_2_CH_3_), 2.72 (dd, *J* = 10.4, 7.2 Hz, 1H, H-2), 3.23 (dd, *J* = 7.6, 2.5 Hz, 1H, H-5), 3.27 (dd, *J* = 10.8, 5.6 Hz, 1H, H-2), 3.45 (d, *J* = 13.6 Hz, 1H, CH_2_Ph), 3.68 (m, 1H, H-3), 3.71 (s, 3H, OCH_3_), 3.81 (m, 1H, H-4), 4.09–4.21 (m, 3H, CH_2_CH_3_ and CH_2_Ph), 4.24–4.34 (m, 2H, CH_2_CH_3_), 7.09 (t, *J* = 7.6 Hz, 1H, ArH), 7.20 (d, *J* = 8.4 Hz, 2H, ArH), 7.29–7.34 (m, 2H, ArH), 7.43–7.46 (m, 2H, ArH), 7.55–7.58 (m, 2H, ArH), 9.18 (s, 1H, NH); ^13^C NMR (100.6 MHz) δ 16.4 (d, *J* = 5.5 Hz, CH_2_CH_3_), 16.5 (d, *J* = 5.5 Hz, CH_2_CH_3_), 44.2 (d, *J* = 7.5 Hz, C-3), 49.9 (C-4), 52.3 (OCH_3_), 56.6 (d, *J* = 13.5 Hz, C-2), 58.8 (d, *J* = 4.5 Hz, CH_2_Ph), 62.9 (d, *J* = 7.5 Hz, CH_2_CH_3_), 63.8 (d, *J* = 7.0 Hz, CH_2_CH_3_), 63.9 (d, *J* = 169.5 Hz, C-5), 119.5 (2CHAr), 121.0 (C-ipso), 124.1 (CHAr), 128.9 (2CHAr), 130.2 (2CHAr), 131.5 (2CHAr), 137.3 (C-ipso), 138.2 (C-ipso), 168.8 (d, *J* = 4.0 Hz, CO), 173.3 (CO); MS-EI m/z 552 M^+^ (1),417 (100), 383 (12), 322 (13), 296 (21), 264 (12), 236 (19), 171 (83), 90 (19); HRMS C_24_H_31_BrN_2_O_6_P [M + H]^+^ 553.1099; found, 553.1098.

### 3.2. X-Ray Crystallographic Analysis of PIPred02, CPP06

Crystals of **2d** and **5** were obtained from the slow evaporation of methanol solutions. The single-crystal X-ray diffraction data sets were collected at 294 K up to a max 2ϑ of ca. 57° and 50°, respectively, on a Bruker Smart APEX II diffractometer, using monochromatic MoKα radiation λ = 0.71073 Å and 0.3° separation between frames. Data integration was performed using SAINT V6.45A and SORTAV in the diffractometer package [34]. The crystal and collection data and structural refinement parameters are given in Tables S1–S10. The structures were solved by direct methods using SHELXT-2014 and Fourier’s difference methods [35], and refined by least squares on F2 using SHELXL-2014/7 inside the WinGX program environment [36]. Anisotropic displacement parameters were used for non-H atoms (Tables S4 and S9) and the H-atoms were positioned in calculated positions and refined by riding on their parent atoms. Atom coordinates are given in Tables S2 and S7 and bond distances and angles in Tables S3 and S8. Hydrogen bonds are detailed in Tables S5 and S10.

Here, **2d** crystallized in the orthorhombic Pca21 space group as chloride (Ortep representation in Scheme 2). Two independent groups constitute the asymmetric unit (A and B). Both pyrrolidinium rings present a twisted conformation on C2-C3 (in A) and on C22-C23 (in B), while the phenyl groups are flat. The stereocenters C2 and C3 exhibit chiralities R and S, respectively (in A). The other molecule shows opposed chirality, S and R for C22 and C23, respectively (in B). So, although the space group is chiral, as well as the crystal, as is clearly shown by the Flack parameter (0.07 (7)), the two molecules being of opposed chirality indicates that this chiral crystal has a racemic composition (!). Molecules of the same type are concatenated along the **a** axis, either through O1···N1’ (+)···Cl1 (-)···N7 (in A) or O4···N21’ (+)···Cl2 (-)···N27 (in B) hydrogen bonds (Table S5 of the **2d** tables), where the protonated N1’ (+) and N21’ (+), respectively, correspond to the neighboring molecule in the (100) direction. So, molecules pack in separated chains, of either A or B type.

Compound **5** crystallizes in the monoclinic C2/c space group (Ortep in Scheme 3). Two independent molecules constitute the asymmetric unit (C and D). The pyrrolidine rings present twisted conformations on C3-C4 (in C) and on C13-C14 (in D), while the phenyl groups are flat. The stereocenters C3, C4, and C5 exhibit chiralities R, S, and S, respectively (in C); similarly, in the other molecule, C13, C14 and C15 show the same chirality scheme, R, S, and S, respectively (in D). The crystal is racemic, even if both molecules have the same chirality, because of the centrosymmetry of the space group. Molecules are packed in dimers through the N2···O7 and N6···O1 hydrogen bonds (see Table S10 for **5**). So, the asymmetric unit consists of a C:::D dimer.

Crystallographic data for the reported structures has been deposited in the Cambridge Crystallographic Data Centre as a supplementary publication, CCDC No.2442162 (for **2d**), and CCDC No. 2442161 (for **5**). Copies of this information may be obtained free of charge from The Director, CCDC, 12 Union Road, Cambridge CB2 1EZ, UK. Fax: þ44 1223 336 033.

Email: data_request@ccdc.cam.ac.uk. Web page: http://www.ccdc.cam.ac.uk (8 April 2025). 

### 3.3. Binding Studies

#### 3.3.1. Preparation of Cellular Membranes

Human brain samples were obtained via autopsy in the Basque Institute of Legal Medicine, Bilbao, Spain. Samples from the prefrontal cortex (Brodmann’s area 9) were dissected at the time of autopsy and immediately stored at −70 °C until assay. The study was developed in compliance with policies of research and ethical review boards for postmortem brain studies.

To obtain cellular membranes (P2 fraction), the different samples were homogenized using an ultraturrax in 10 volumes of homogenization buffer (0.25 M sucrose, 5 mM Tris–HCl, pH 7.4). The crude homogenate was centrifuged for 5 min at 1000× *g* (4 °C) and the supernatant was centrifuged again for 10 min at 40,000× *g* (4 °C). The resultant pellet was washed twice in 5 volumes of homogenization buffer and recentrifuged in similar conditions. Protein content was measured according to the method of Bradford using BSA as standard.

#### 3.3.2. Competition Binding Assays

The pharmacological activity of the compounds was evaluated through competition binding studies against the I_2_-IR selective radioligand [^3^H]2-BFI or the α_2_-adrenergic receptor selective radioligand [^3^H]RX821002. Specific binding was measured in 0.25 mL aliquots (50 mM Tris-HCl, pH 7.5) containing 100 µg of membranes, which were incubated in 96-well plates either with [^3^H]2-BFI (2 nM) for 45 min at 25 °C or with [^3^H]RX821002 (1 nM) for 30 min at 25 °C, in the absence or presence of the competing compounds (10^−12^ to 10^−3^ M, 10 concentrations).

Incubations were terminated by separating free ligand from bound ligand by rapid filtration under vacuum (1450 Filter Mate Harvester, PerkinElmer) through GF/C glass fiber filters. The filters were then rinsed three times with 300 μL of binding buffer, air-dried (120 min), and assessed for radioactivity by liquid scintillation spectrometry using a MicroBeta TriLux counter (PerkinElmer). Specific binding was determined and plotted as a function of the compound concentration. Nonspecific binding was determined in the presence of idazoxan (10^−5^ M), a compound with well-established affinity for I_2_-IR and α_2_-adrenergic receptors, in [^3^H]2-BFI and [^3^H]RX821002 assays. To obtain the inhibition constant (*K*_i_), analyses of competition experiments were performed by nonlinear regression using the GraphPad Prism program. *K*_i_ values were normalized to p*K*_i_ values. I_2_-IR/α_2_-AR selectivity index was calculated as the antilogarithm of the difference between p*K*_i_ values for I_2_-IR and p*K*_i_ values for α_2_-AR.

## 4. Conclusions

To sum up, we have proposed a new method for accessing diversely substituted phosphoprolines, a key heterocyclic nucleous endowed with huge biological potential, by the description of a methodology that permits the synthesis of 2,2,3-trisubtituted and 2,2,3,3-tetrasubstituted (pyrrolidine-2-yl)phosphonates. The new compounds were characterized from the stereochemical point of view by X-ray crystallography analysis. Two selected compounds, **2e** and **4e**, that share structural moieties, the 3Cl,4-FPh substituent in the nitrogen atom and the α-phenylphosphonate, with the I_2_-IR ligands previously reported by us showed good in vitro BBB penetration. While the structural modifications in **2e** proved deleterious for the affinity with I_2_-IR, the presence of the methyl ester functional group in the 4 position in **4e** contributed to a compound that did not outperform the previous I_2_-IR ligands proposed by us, but which had affinity and selectivity values for I_2_-IR in the range of clinical candidates CR4056 and BU99008 in human tissues. Therefore, **4e** has emerged as a promising compound for further pharmacological studies.

## Figures and Tables

**Table 1:** Significant ^1^H and ^13^C-NMR data (ppm, multiplicity and coupling constants, Hz) of new compounds **2a**–**2g**.

	^1^H-NMR Data						^13^C-NMR Data			
Comp.	H2	H3	H4	H4	H5	H5	C2	C3	C4	C5
**2a**	3.63 t ^1^ 7.5	3.26 ddd ^2^ 24.0, 16.0, 8.0	2.06 m ^3^	2.19–2.29 c.s. ^4^	2.99–3.12 c.s.		57.8 d ^5^ 165.0	47.5 d 2.0	31.2 d 7.0	48.4 d 9.0
**2b**	3.46 t 7.5	3.75 m	1.97 m	2.13–2.22 c.s.	2.91–3.07 c.s.		58.5 d 165.0	48.2	30.9 d 6.5	48.4 d 9.5
**2c**	-	3.58 ddd 13.6, 7.6, 6.0	2.43–2.53 c.s.		3.48 ddd 11.2, 8.4, 6.8	3.69 ddd 11.2, 8.4, 6.8	64.4 d 159.0	48.8	27.8 d 4.0	45.2 d 3.0
**2d**	-	4.32 dd ^6^ 10.8, 7.6	2.42 m	2.83 m	3.81–3.89 c.s.		72.8 d 149.5	49.9	28.3	45.8
**2e**	-	4.30 dd 10.4, 7.6	2.43 m	2.83 m	3.79–3.91 c.s.		72.7 d 150.0	50.0	28.2	45.8
**2f**	-	4.28 dd 10.5, 7.5	2.49 m	2.84 m	3.74–3.98 c.s.		74.2 d 149.5	51.6	29.7	47.2
**2g**	-	4.06–4.17 c.s.	2.25 m	2.73 m	3.77–3.88 c.s.		72.5 d 149.5	49.1	28.1	45.9

^1^ t = triplet; ^2^ ddd = doublet of doublets of doublets; ^3^ m = multiplet; ^4^ c.s. = complex signal; ^5^ d = doublet and ^6^ dd = doublet of doublets.

**Table 2:** Significant ^1^H and ^13^C-NMR data (ppm, multiplicity and coupling constants, Hz) of new compounds **4a**–**4e** and **5**.

	^1^H-NMR Data					^13^C-NMR Data			
Comp.	H2	H2	H3	H4	H5	C2	C3	C4	C5
**4a**	3.22–3.33 c.s. ^1^		2.53–3.67 c.s.	2.53–3.67 c.s.	2.53–3.67 c.s.	52.1 d ^2^ 11.5	48.2 d 7.5	51.2 d 2.5	58.9 d 163.0
**4b**	3.25 m ^3^	3.31 m	3.48–3.65 c.s.	3.48–3.65 c.s.	3.48–3.65 c.s.	52.1 d 12.0	47.6 d 8.0	51.1 d 2.2	58.8 d 162.0
**4c**	3.19–3.28 c.s.		3.74 m	3.34 ddd ^4^ 19.0, 8.0, 6.8	3.45 d 8.8	51.9 d 10.5	48.5 d 7.0	50.0 d 2.0	59.0 d 164.0
**4d**	3.31 t ^5^ 9.5	3.62–3.81 c.s.	3.62–3.81 c.s.	3.95–4.05 c.s.	-	49.2 d 7.0	46.2 d 7.5	57.2 d 4.0	68.7 d 158.0
**4e**	3.29 m	3.64–3.70 c.s.	3.64–3.70 c.s.	3.94–4.07 c.s.	-	49.3 d 7.0	45.7 d 7.5	57.2 d 3.5	68.4 d 159.0
**5**	2.72 dd ^6^ 10.4, 7.2	3.27 dd 10.8, 5.6	3.68 m	3.81 m	3.23 dd 7.6, 2.5	56.6 d 13.5	44.2 d 7.5	49.9	63.9 d 169.5

^1^ c.s. = complex signal; ^2^ d = doublet; ^3^ m = multiplet; ^4^ ddd = doublet of doublets of doublets; ^5^ t = triplet and ^6^ dd = doublet of doublets.

**Table 3:** I_2_-IR and α_2_-AR binding affinities (p*K*_i_) of two clinical candidates CR4056 and BU99008, and four previously reported compounds, B06, B06red, BIN05 and PIP01 (**1e**), and new compounds **2e** and **4e**. Table adapted from reference [11].

Entry	Compd.	[^3^H]-2-BFI, I_2_ One Site ^b^ I_2_ Two Sites H/L; (p*K*_iH_/p*K*_iL_) High Affinity Site %	[^3^H]RX821002, α_2_	Selectivity I_2_/α_2_^a^
1	CR4056 ^c^	7.72 ± 0.31/5.45 ± 0.15 29 ± 6	2.65 ± 1.24	117,490
2	BU99008 ^c^	6.89 ± 0.21/3.82 ± 0.30 51 ± 6	4.37 ± 0.17	331
3	B06 ^c^	8.56 ± 0.32 8.61 ± 0.28/4.29 ± 0.20 37 ± 4	6.27 ± 0.56	195
4	B06red ^d^	6.88 ± 0.29 9.81 ± 0.21/< 3 50 ± 4	6.66 ± 0.49	1.7
5	BIN05 ^d^	3.89 ± 0.19 8.18 ± 0.43/3.56 ± 0.22 21 ± 3	5.51 ± 0.24	-
6	PIP01 ^c^ (**1e**)	9.98 ± 0.74	9.43 ± 0.22	3
7	**2e**	4.16 ± 0.15	4.07 ± 0.20	1.2
8	**4e**	7.99 ± 0.39	3.22 ± 0.15	58,884

^a^ Selectivity I_2_-IR/α_2_-AR expressed as the antilog (p*K*_i_ I_2_-IR p*K*_i_ α_2_-AR). ^b^ The best fit of the data for CR4056, BU99008, B06, B06red and BIN05 was to a two-site binding model of binding with high p*K*_i_ (p*K*_iH_) and low p*K*_i_ (p*K*_iL_) affinities for both binding sites, respectively. ^c^ Values published in [11]. ^d^ Values published in [9].

## Data Availability

Data are contained within the article and Supplementary Materials.

## Supplementary Materials

The following supporting information can be downloaded at: https://www.mdpi.com/article/10.3390/molecules30092078/s1, ^1^H-NMR and ^13^C-NMR spectra of new compounds, X-ray crystallographic data for 2d and 5, in vitro Blood–Brain Barrier Permeation Assay, Molecular Formula Strings (SMILES). Ref. [37] is cited in the Supplementary Materials.

## Abbreviations

ADME-Tox, absorption, distribution, metabolism, excretion and toxicity; α_2_-AR, α_2_-adrenergic receptor; CH_3_CN, acetonitrile; EtOH, ethanol; EtOAc, ethyl acetate; HRMS, high-resolution mass spectrometry; MeOH, methanol; NaBH_3_CN, sodium borohydride; NMR, Nuclear Magnetic Resonance; PAMPA, parallel artificial membrane permeability assay; rt, room temperature; SAMP8, senescence accelerated mouse-prone 8; NaOH, sodium hydroxide; THF, tetrahydrofurane.
