# Supplementary material for: Synthesis of Diversely Substituted Diethyl (Pyrrolidin-2-Yl)Phosphonates
Source: Molecules. 2025 May 7;30(9):2078. doi: 10.3390/molecules30092078 (PMC12073250; doi:10.3390/molecules30092078)
Supplement: Supplementary file 1 [file molecules-30-02078-s001.zip › molecules-3606082-supplementary.pdf]

molecules

MDPI

Supplementary Materials

Synthesis of Diversely Substituted Diethyl (Pyrrolidin-2-Yl)Phosphonates

Andrea Bagán [1,2], Alba López-Ruiz [1,2], Sònia Abás [1], Elies Molins [3], Belén Pérez [4], Itziar Muneta-Arrate [5], Luis F. Callado [5,6,7] and Carmen Escolano [1,2,*]

1 Laboratory of Medicinal Chemistry, Department of Pharmacology, Toxicology and Medicinal Chemistry, Faculty of Pharmacy and Fo Sciences, University of Barcelona, Av. Joan XXIII, 27-31, 08028 Barcelona, Spain; andreabaganp@gmail.com (A.B.); alopezru@ub.edu (A.L.-R.); soniaabas88@gmail.com (S.A.)
2 Institute of Biomedicine, University of Barcelona, 08028 Barcelona, Spain
3 Institut de Ciència de Materials de Barcelona (CSIC), Campus UAB, 08193 Cerdanyola del Vallés, Spain; elies.molins@icmab.es
4 Department of Pharmacology, Therapeutic and Toxicology, Autonomous, University of Barcelona, 08193 Cerdanyola del Vallés, Spain; belen.perez@uab.cat
5 Department of Pharmacology, University of the Basque Country (UPV/EHU), 48940 Leioa, Bizkaia, Spain; itziar.muneta@ehu.eus (I.M.-A.); lfcallado@ehu.eus (L.F.C.)
6 Centro de Investigación Biomédica en Red de Salud Mental, CIBERSAM, 28029 Madrid, Spain
7 BioBizkaia Health Research Institute, 48903 Barakaldo, Spain
* Correspondence: cescolano@ub.edu

CONTENTS

^{1}H-NMR and ^{13}C-NMR spectra S1
X-ray crystallographic data for 2d and 5 .... S15
***In vitro* Blood-Brain Barrier Permeation Assay** S36
Molecular Formula Strings (SMILES) S37
References S39

^{1}H-NMR and ^{13}C-NMR spectra

Diethyl [(2*RS*,3*RS*)-3-(phenylcarbamoyl)pyrrolidin-2-yl]phosphonate, 2a

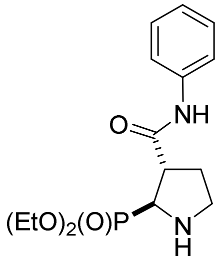

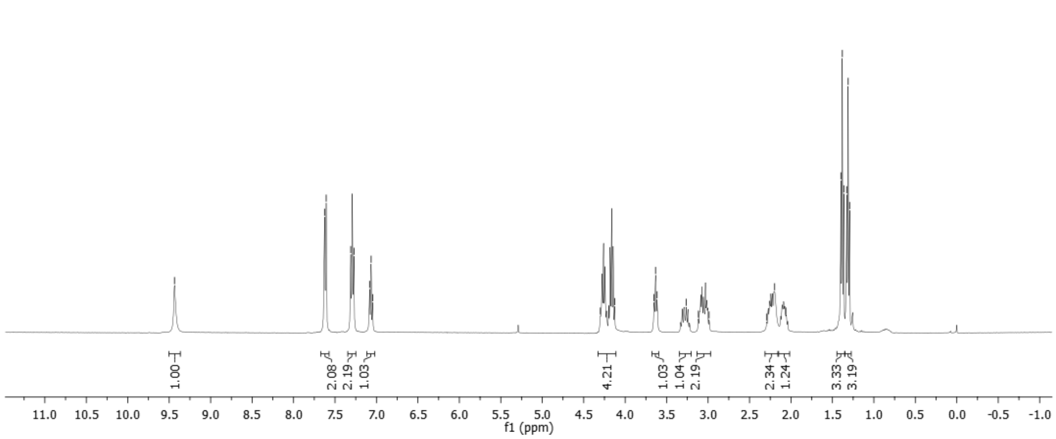

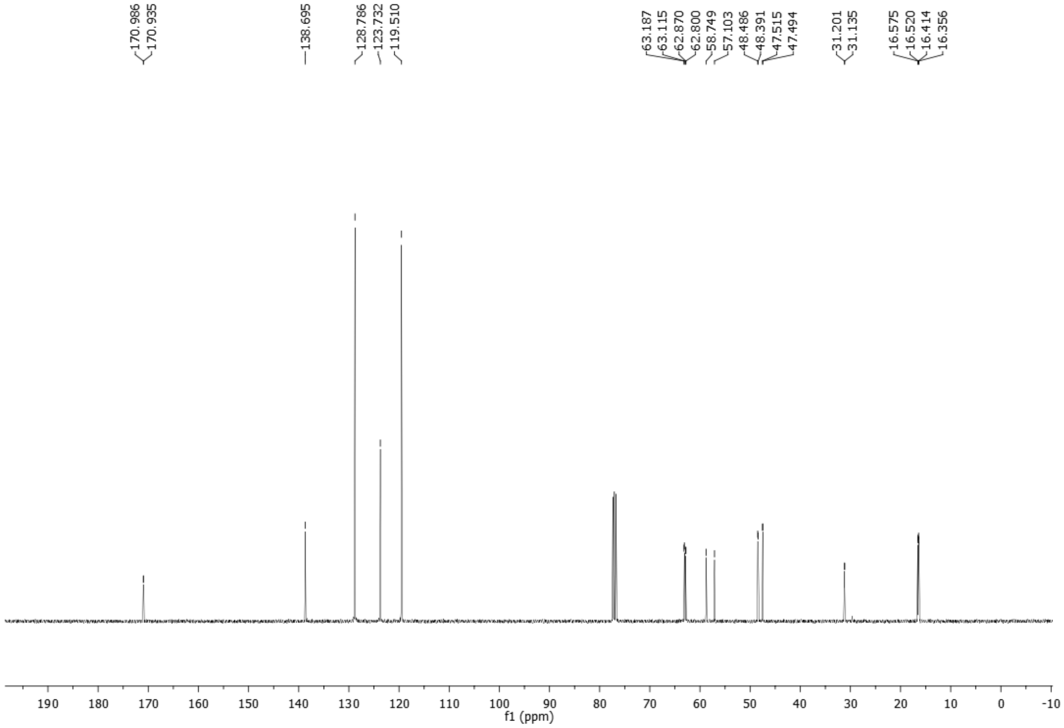

Diethyl [(2*RS*,3*RS*)-3-(cyclohexylcarbamoyl)pyrrolidin-2-yl]phosphonate, 2b

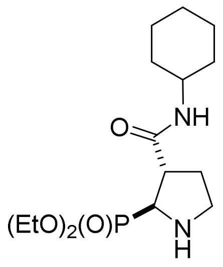

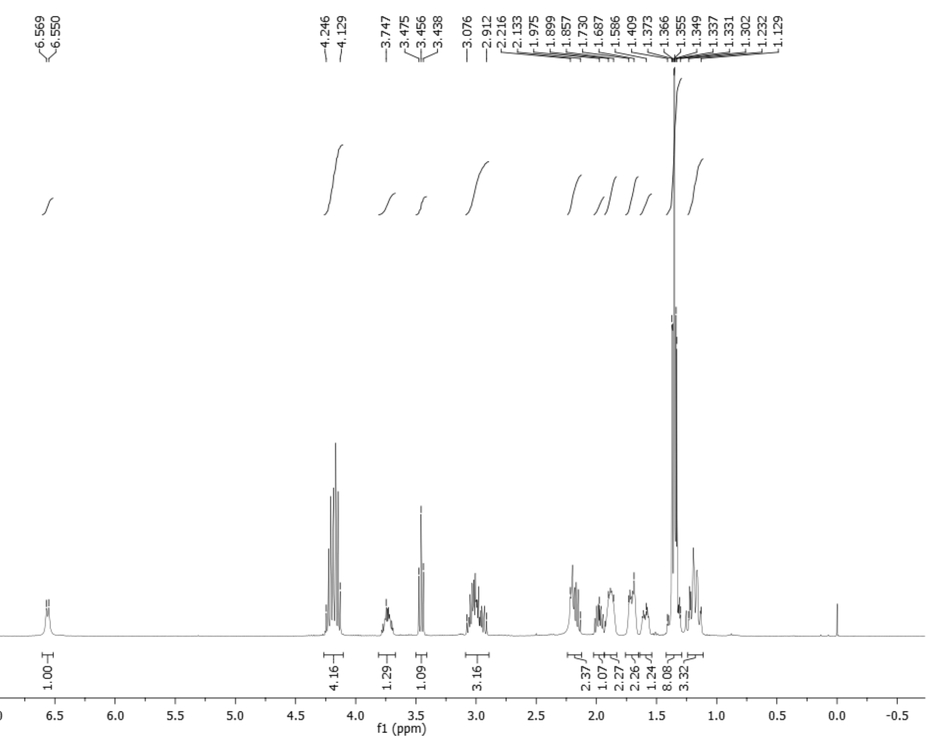

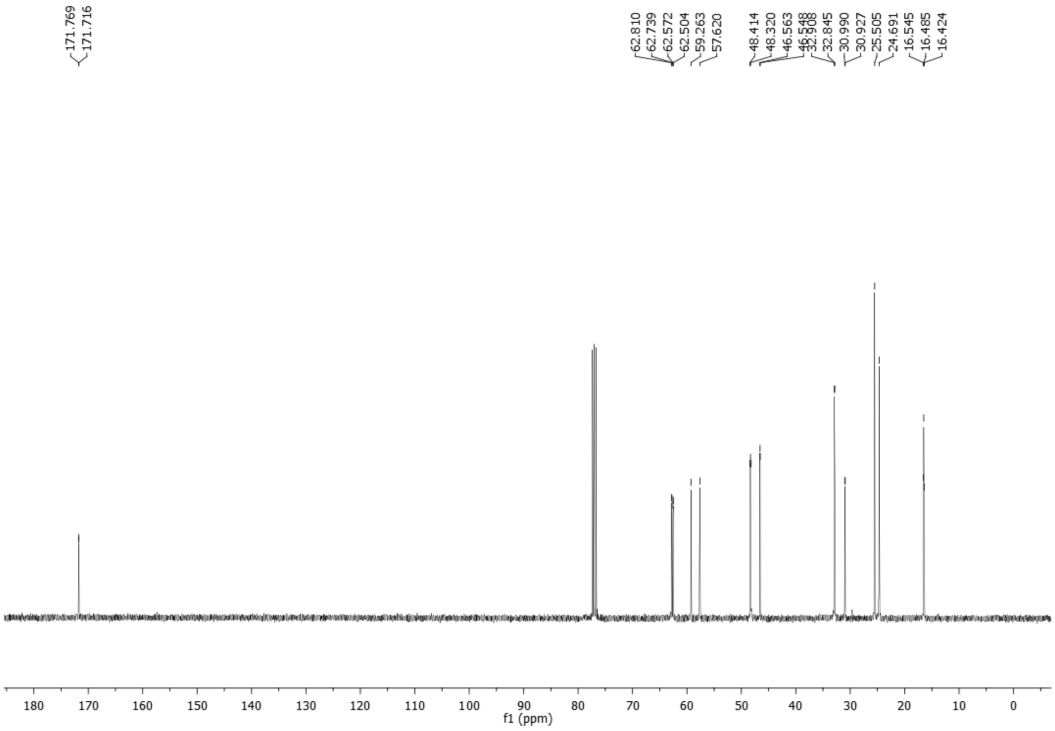

Diethyl [(2*RS*,3*RS*)-2-methyl-3-(phenylcarbamoyl)pyrrolidin-2-yl)phosphonate hydrochloride, 2c

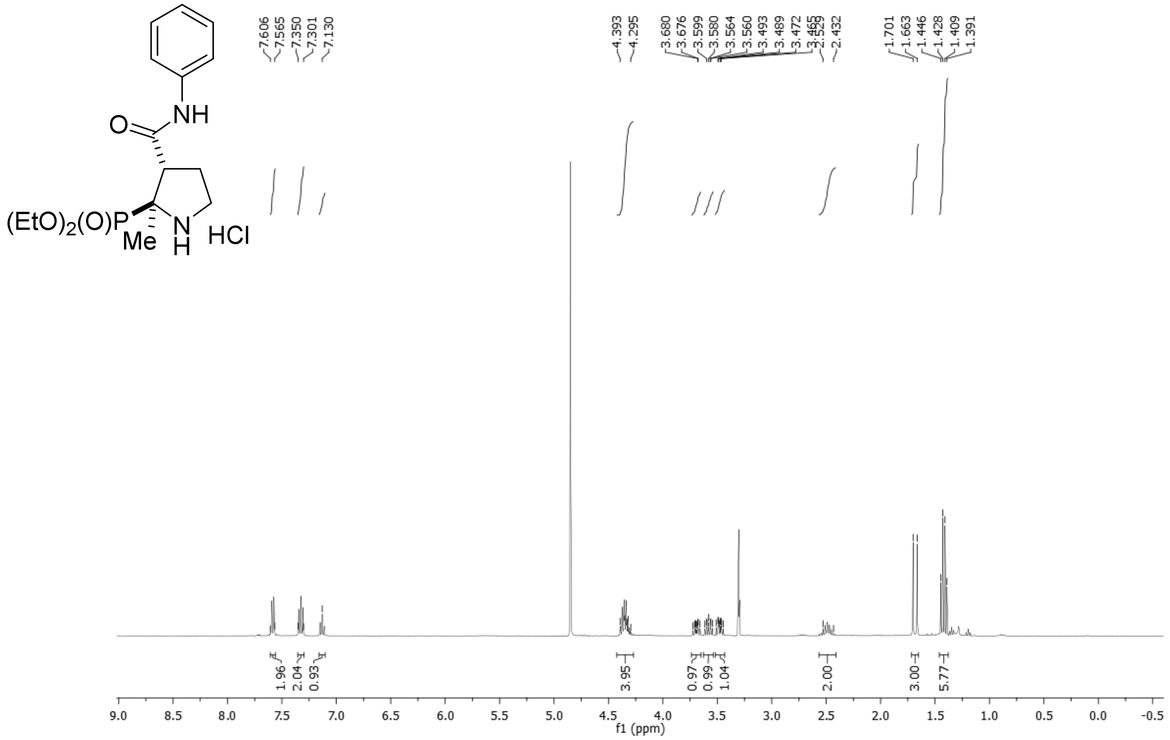

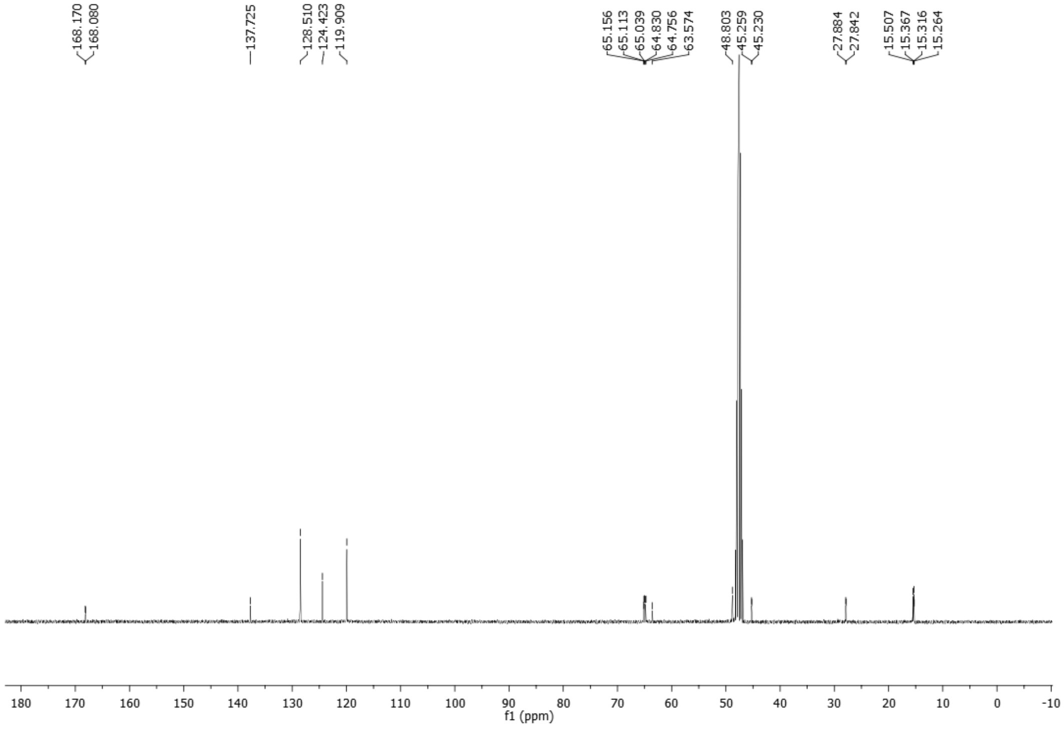

Diethyl[(2*RS*,3*SR*)-2-phenyl-3-(phenylcarbamoyl)pyrrolidin-2-yl]phosphonate hydrochloride, 2d

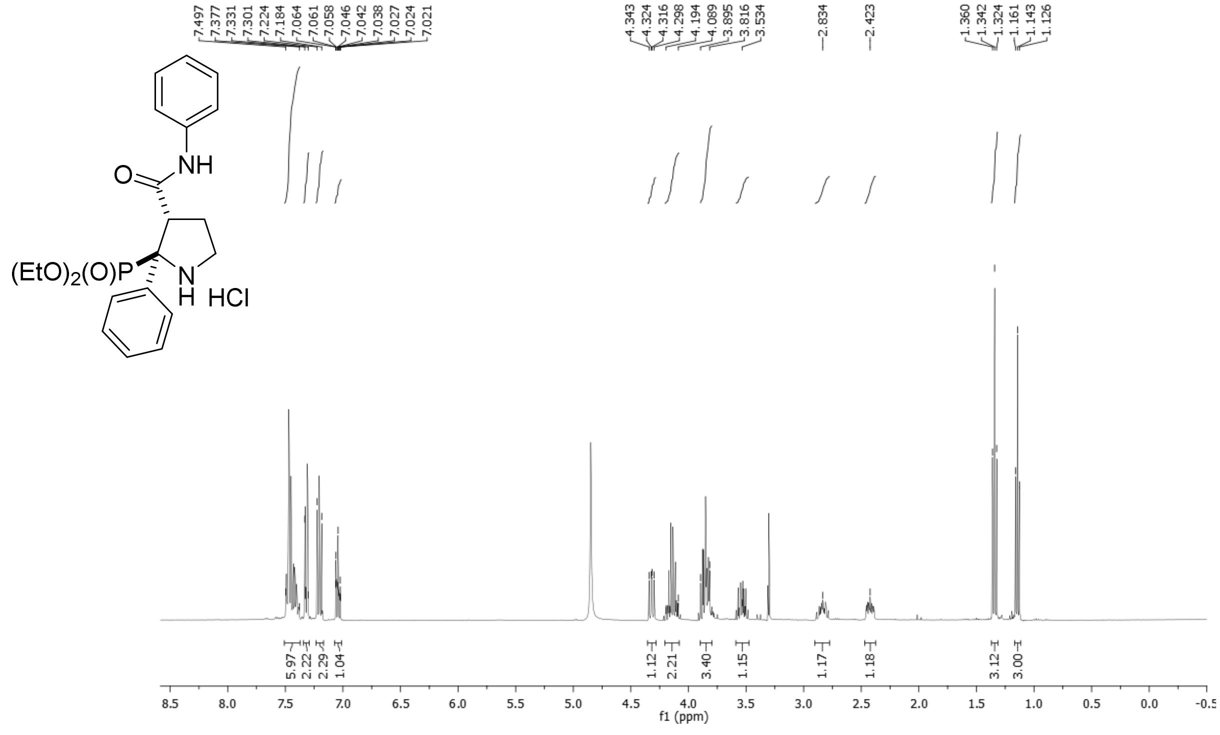

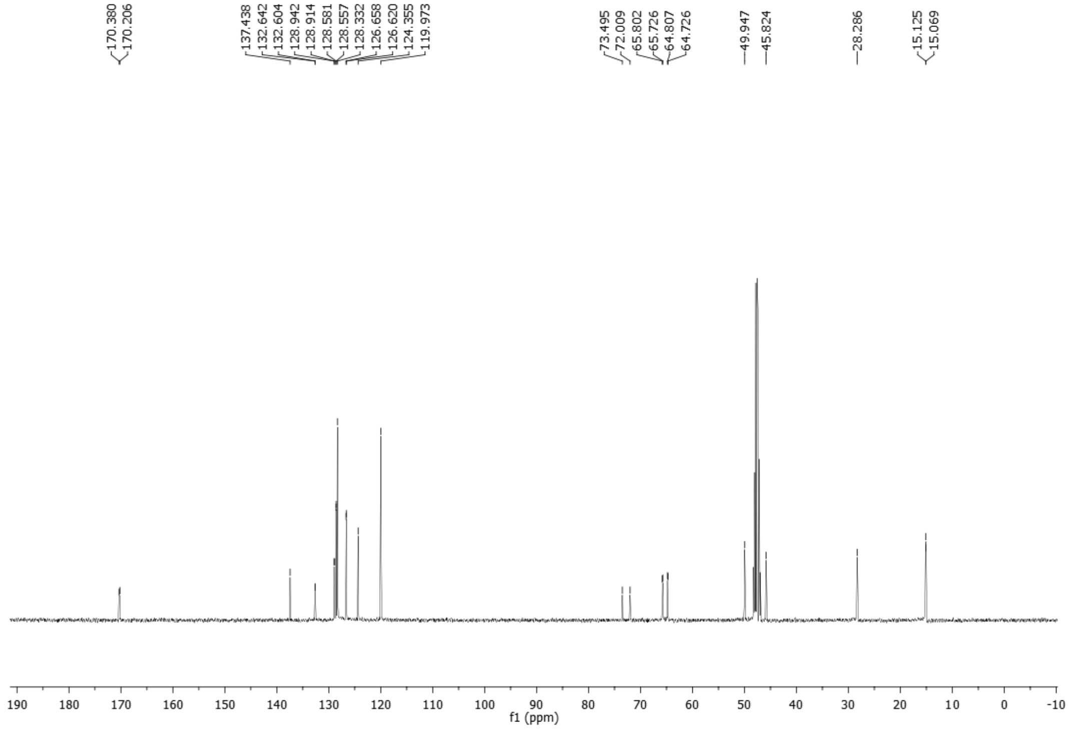

Diethyl [(2*RS*,3*SR*)-3-((3-chloro-4-fluorophenyl)carbamoyl)-2-phenylpyrrolidin-2-yl]phosphonate hydrochloride, 2e

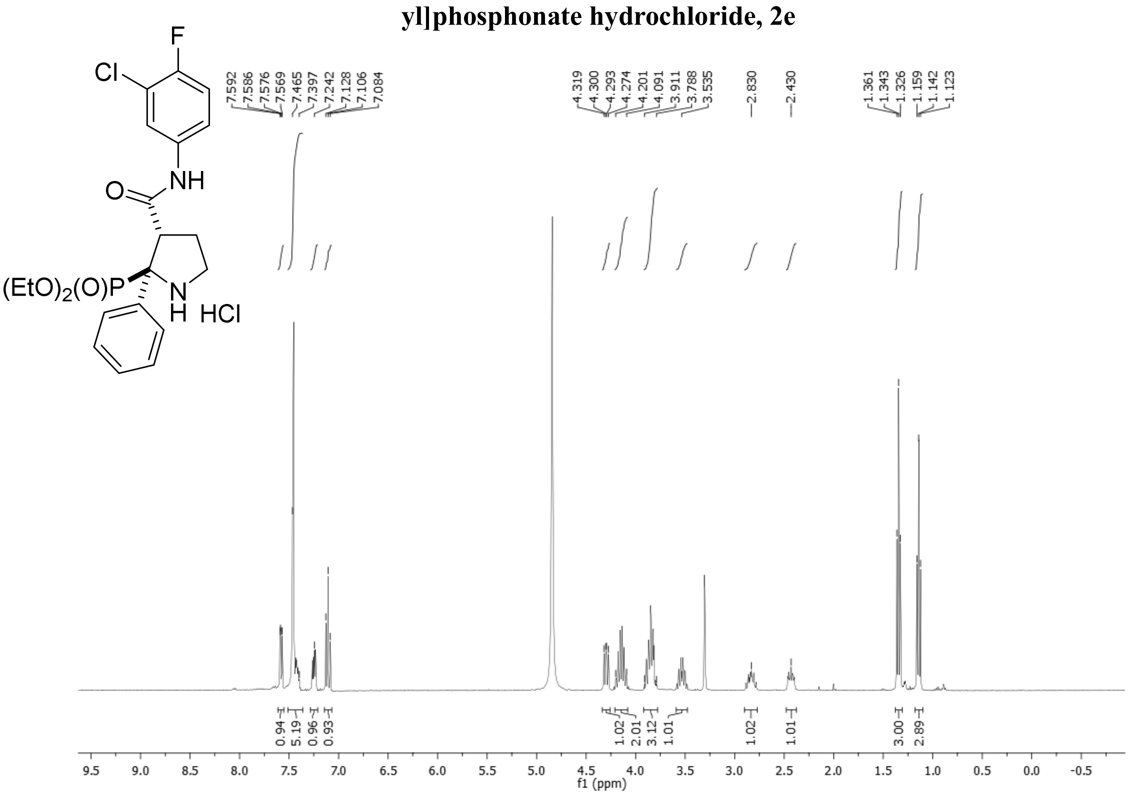

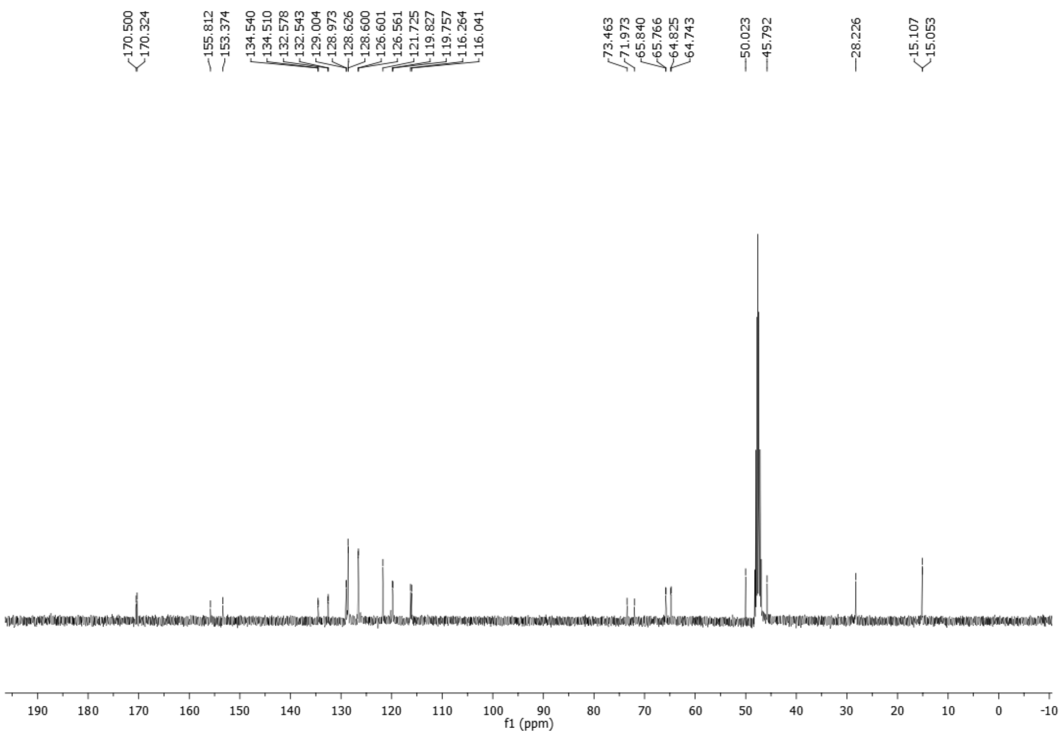

Diethyl ((2*RS*,3*SR*)-2-phenyl-3-((4-(trifluoromethyl)phenyl)carbamoyl)pyrrolidin-2-yl)phosphonate, 2f

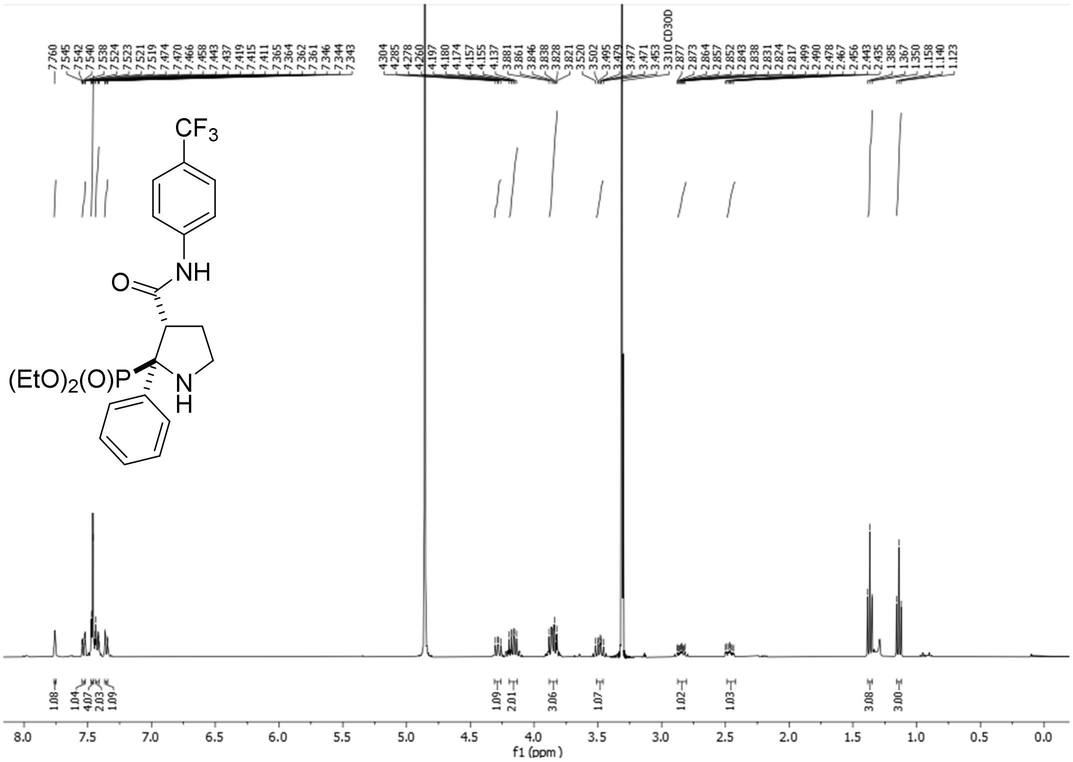

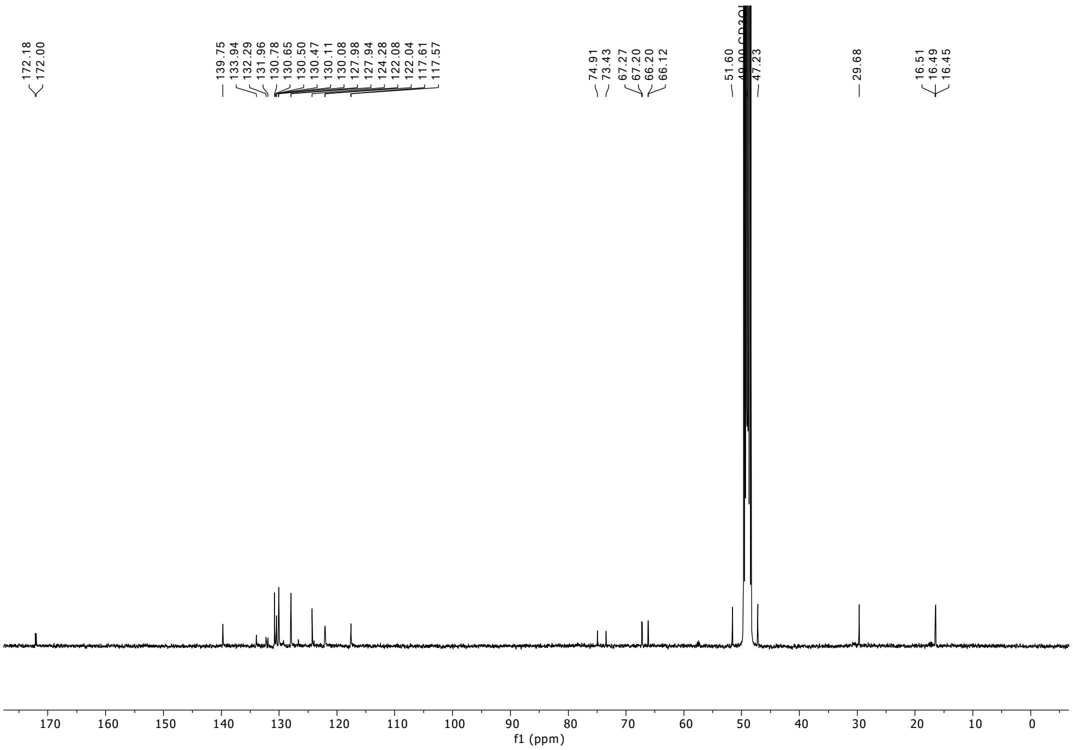

Diethyl [(2*RS*,3*SR*)-3-(cyclohexylcarbamoyl)-2-phenylpyrrolidin-2-yl]phosphonate hydrochloride, 2g

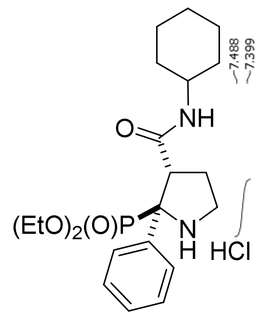

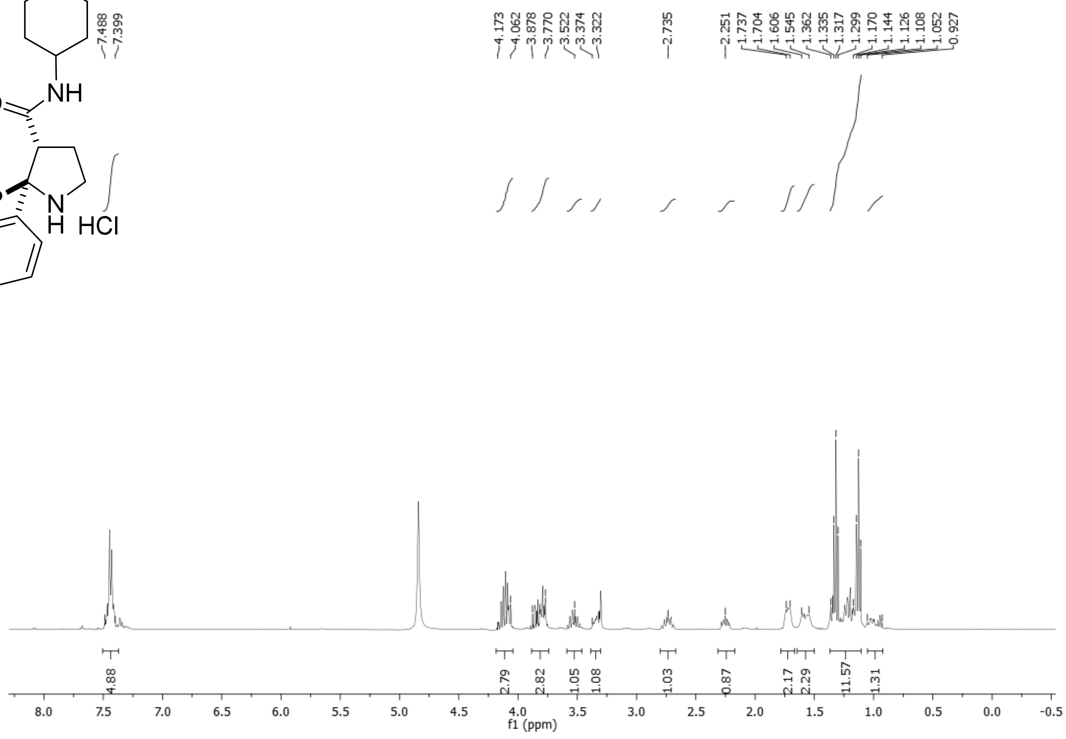

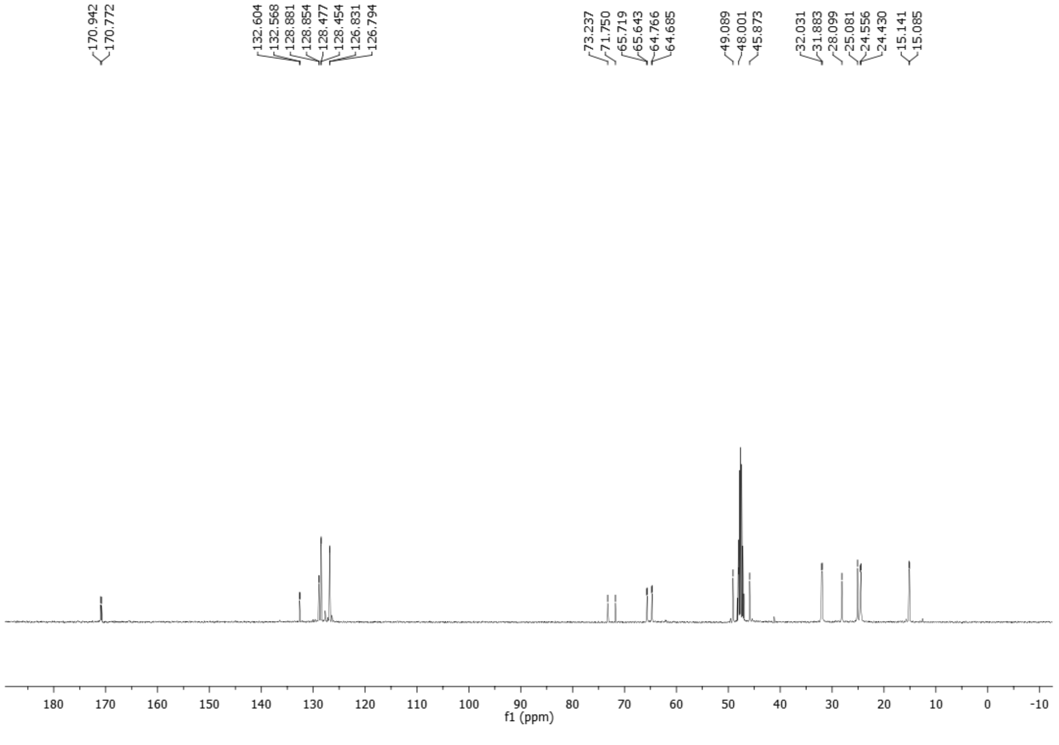

Methyl (3*RS*,4*SR*,5*SR*)-5-(diethoxyphosphoryl)-4-(phenylcarbamoyl)pyrrolidine-3-carboxylate, 4a

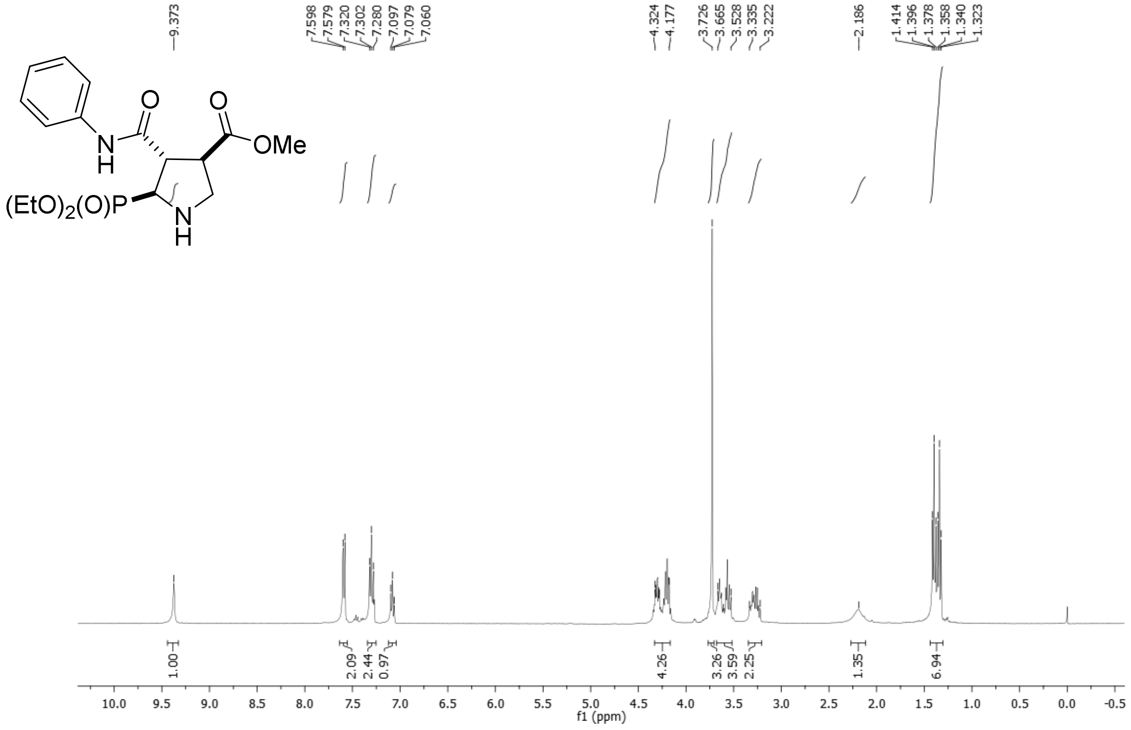

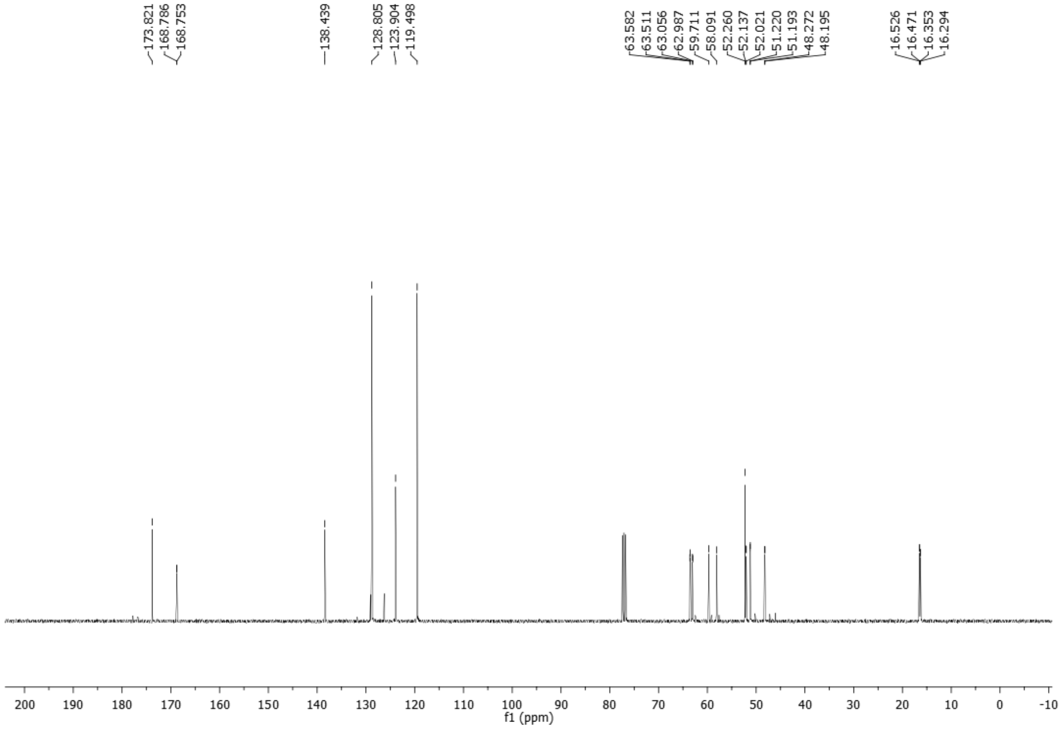

Methyl (3*RS*,4*SR*,5*SR*)-4-((3-chloro-4-fluorophenyl)carbamoyl)-5-(diethoxyphosphoryl)pyrrolidine-3-carboxylate, 4b

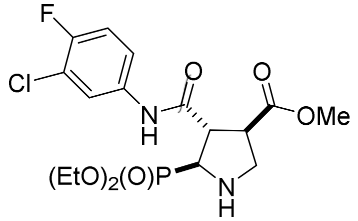

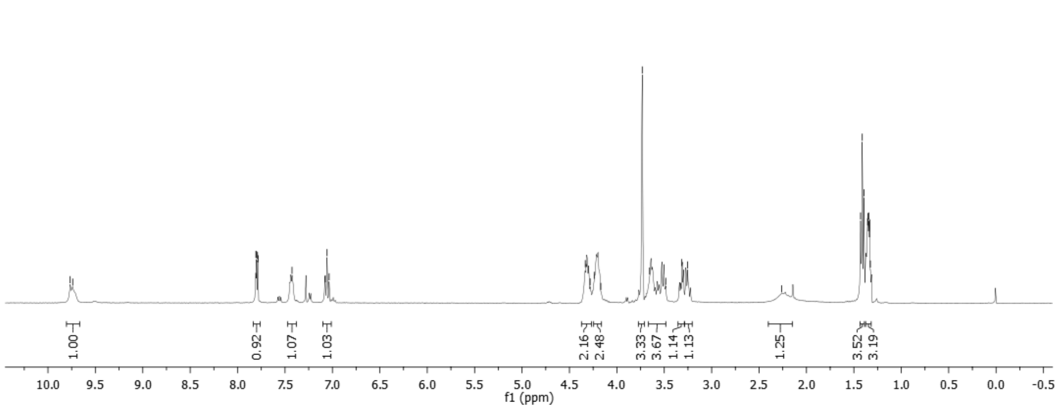

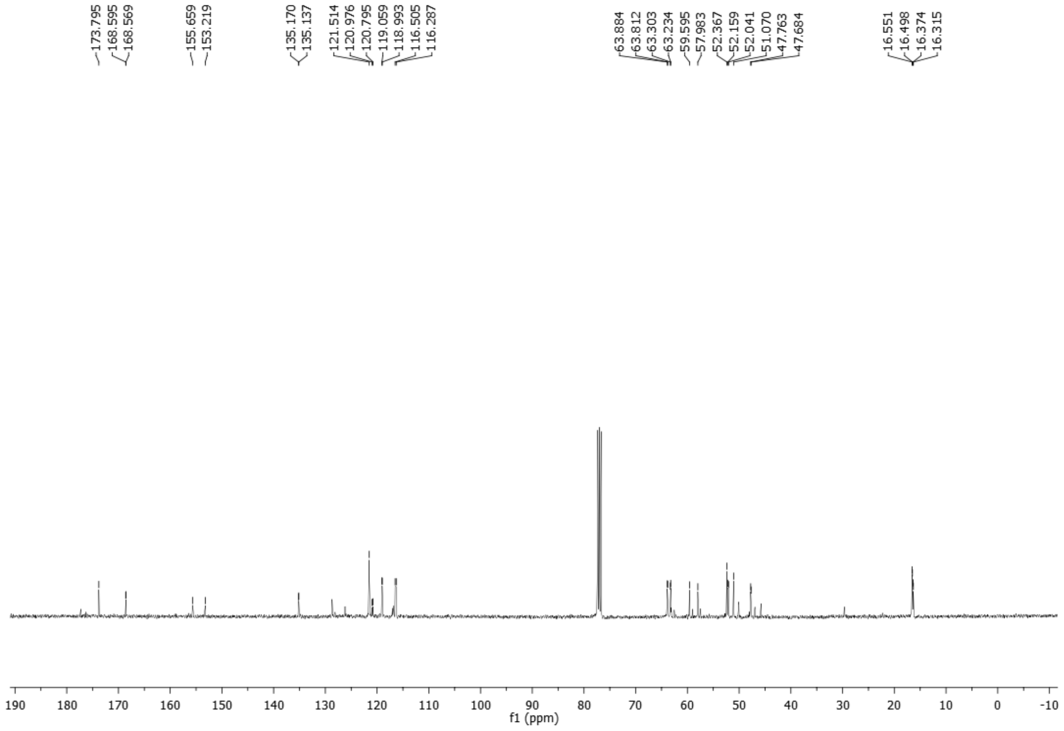

Methyl (3*RS*,4*SR*,5*SR*)-4-(cyclohexylcarbamoyl)-5-(diethoxyphosphoryl)pyrrolidine-3-carboxylate, 4c

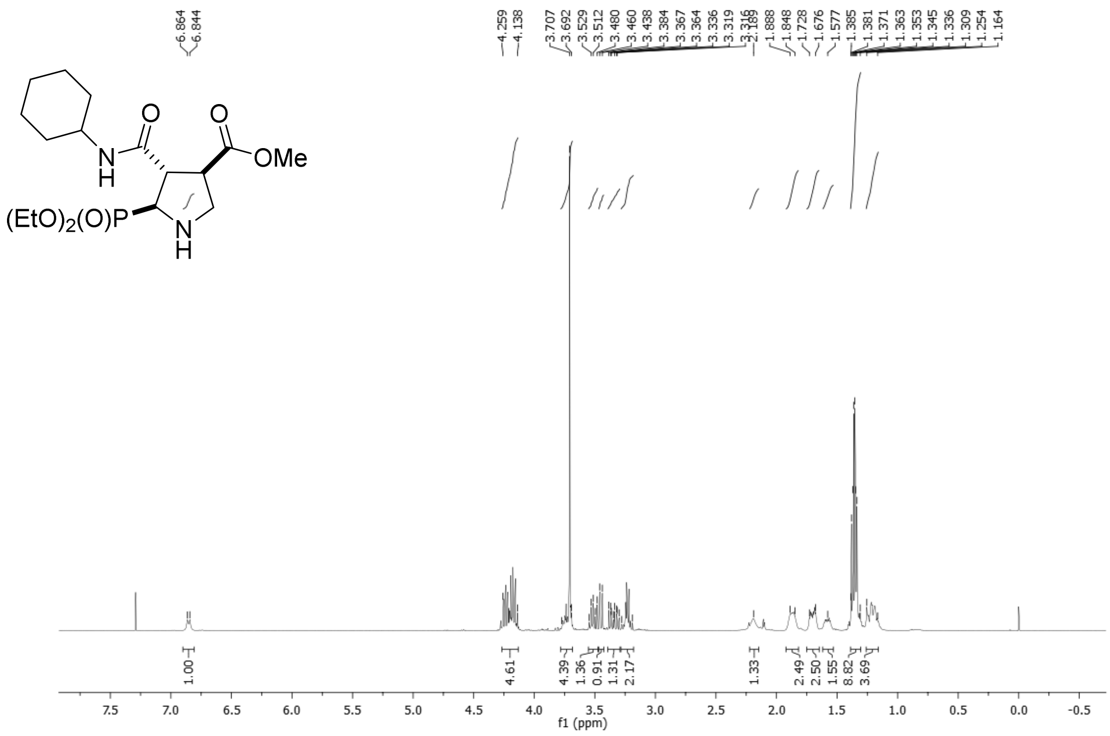

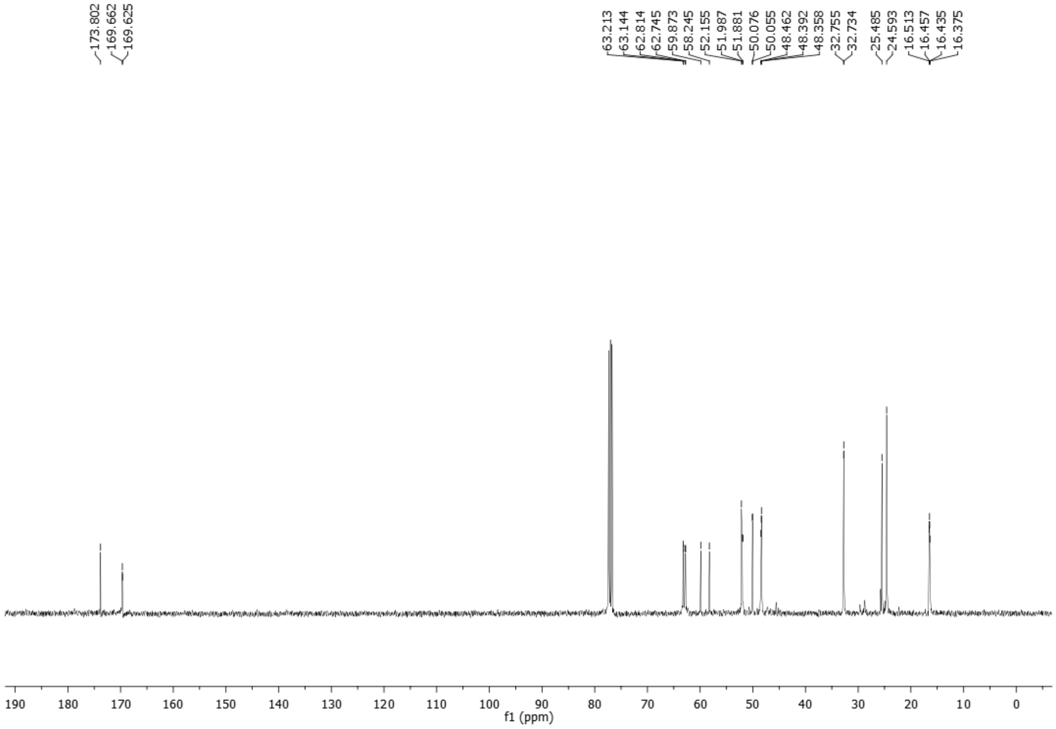

Methyl (3*RS*,4*SR*,5*RS*)-5-(diethoxyphosphoryl)-5-phenyl-4-(phenylcarbamoyl)pyrrolidine-3-carboxylate, 4d

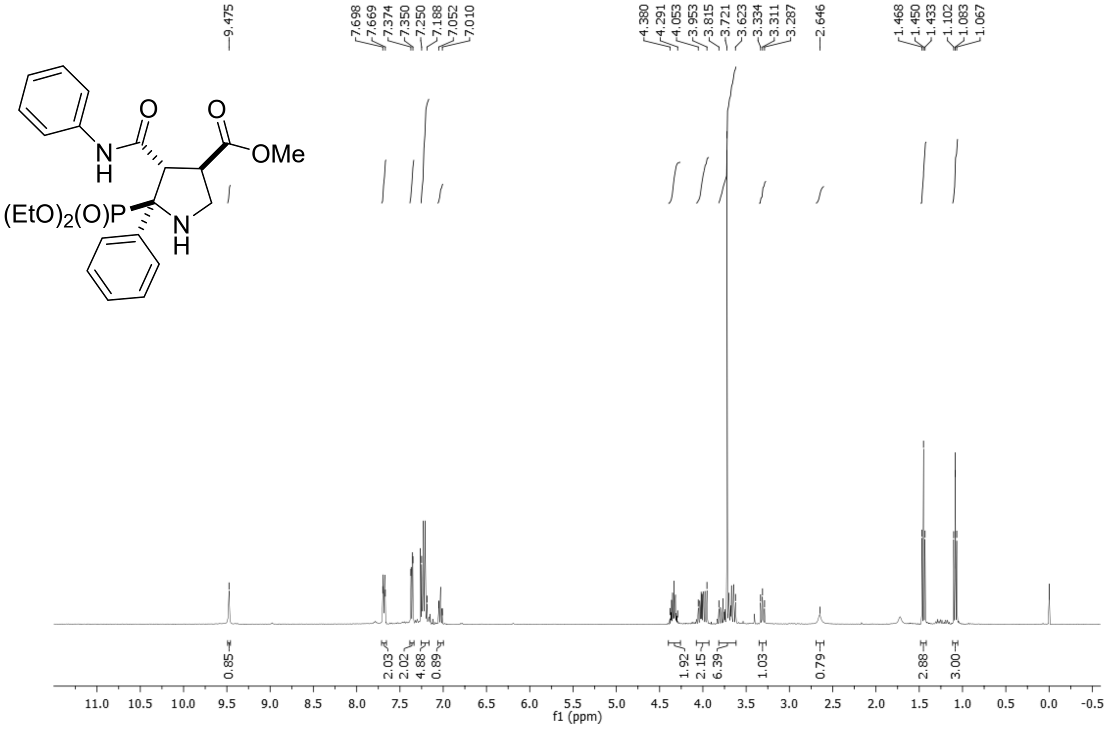

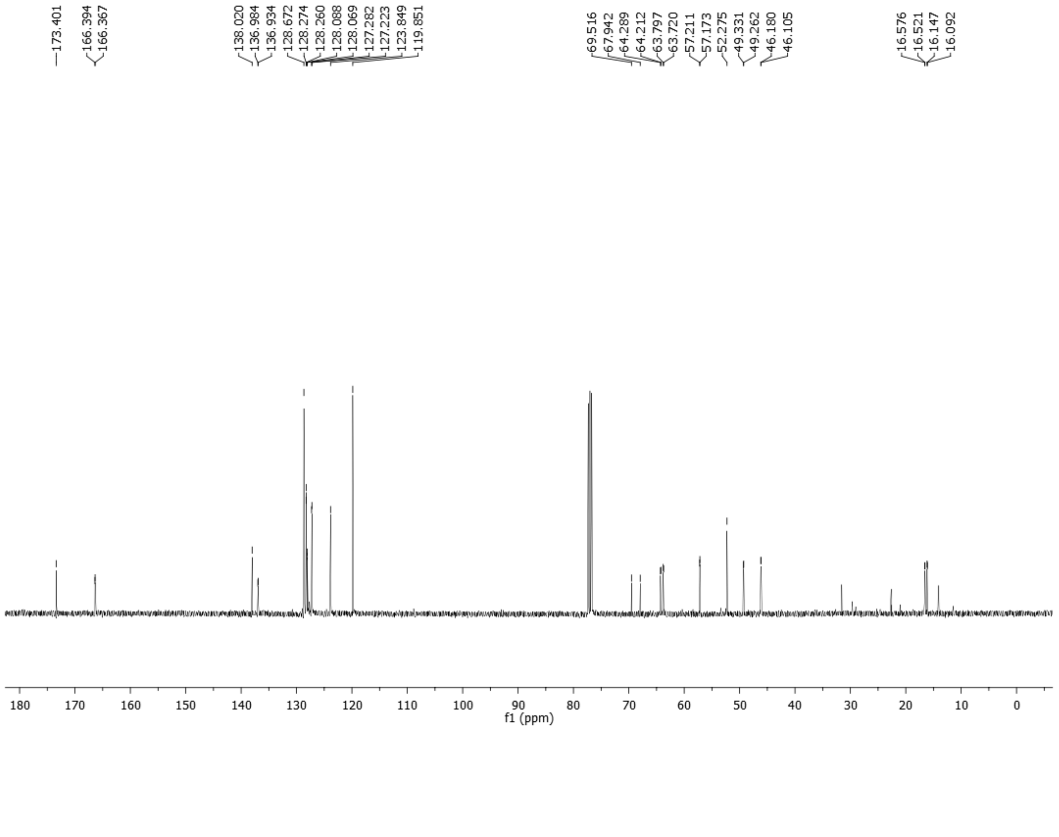

Methyl (3*RS*,4*SR*,5*RS*)-4-((3-chloro-4-fluorophenyl)carbamoyl)-5-(diethoxyphosphoryl)-5-phenylpyrrolidine-3-carboxylate, 4e

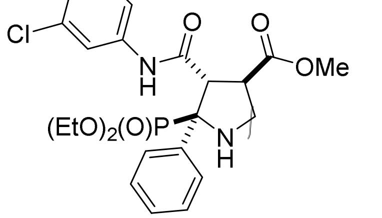

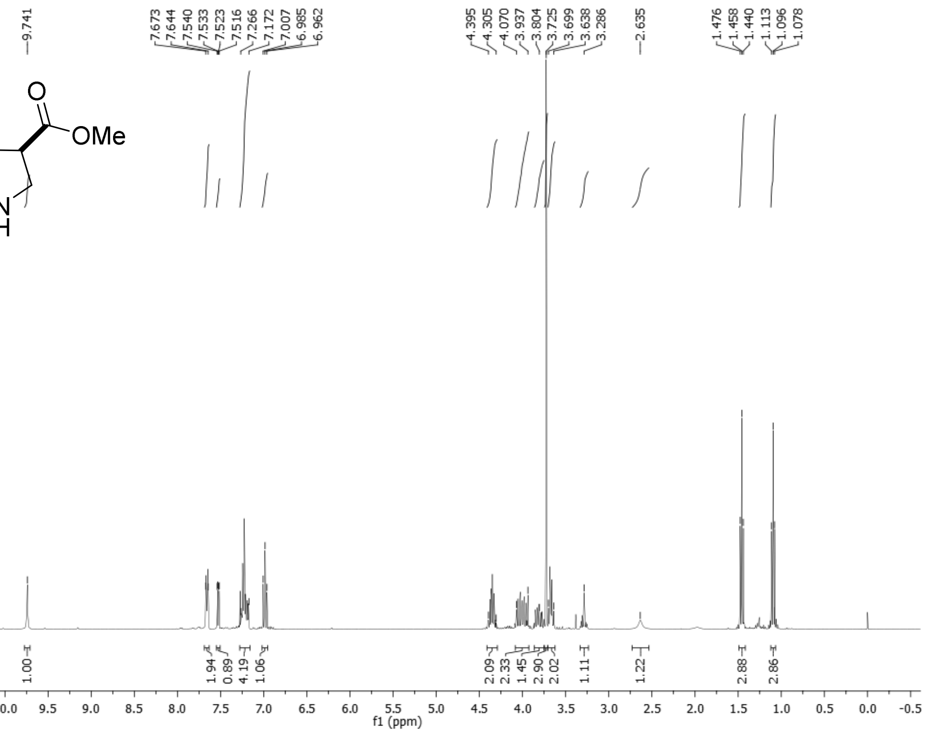

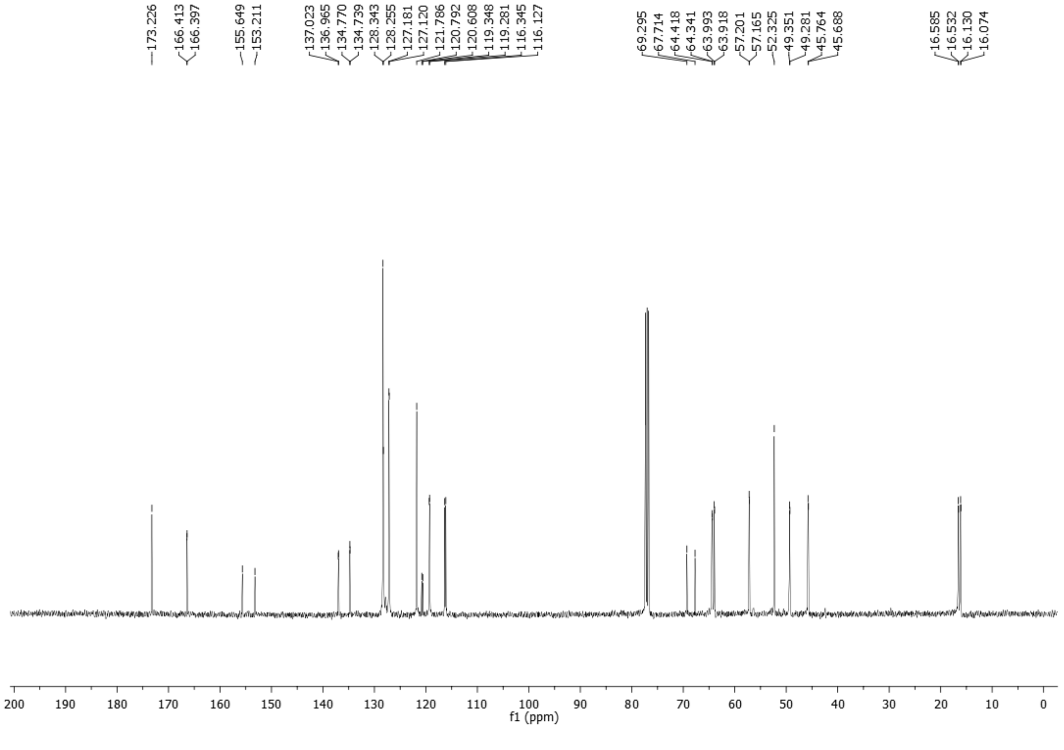

Methyl (3*RS*,4*SR*,5*SR*)-1-(4-bromobenzyl)-5-(diethoxyphosphoryl)-4-(phenylcarbamoyl)pyrrolidine-3-carboxylate, 5

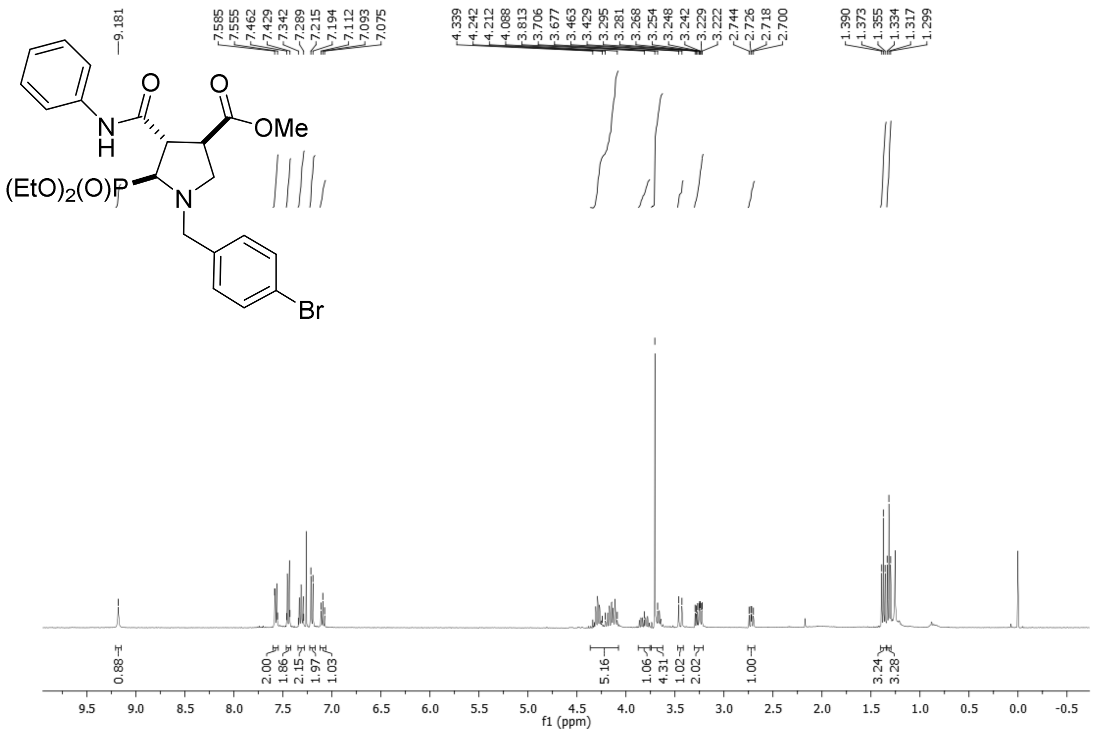

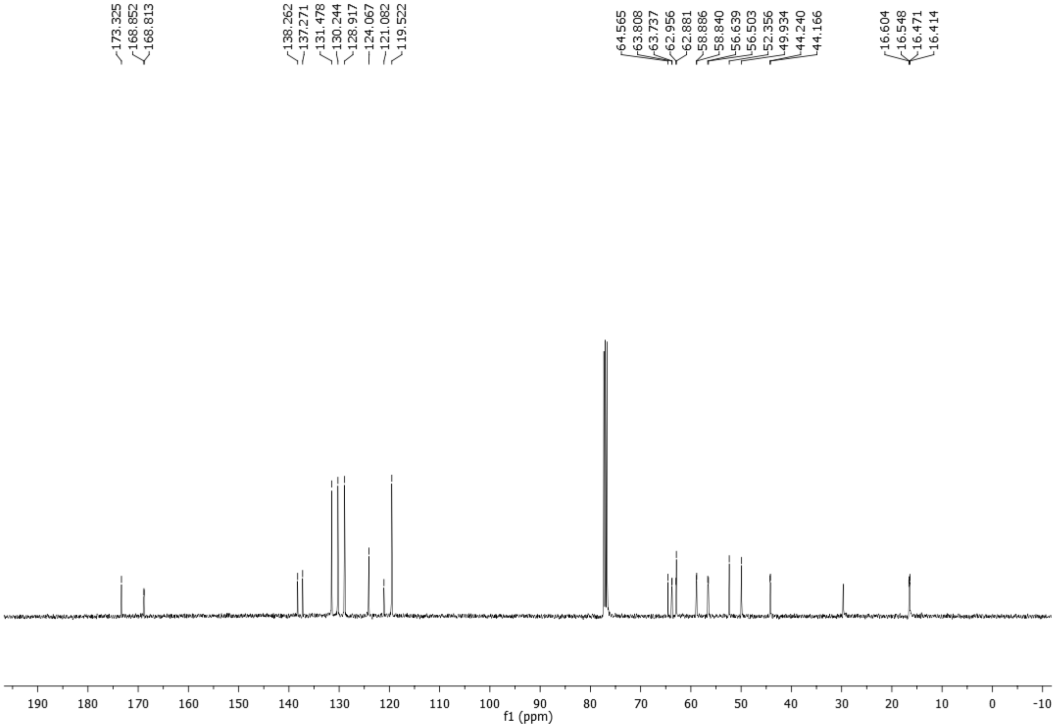

N,5-diphenyl-3,4-dihydro-2*H*-pyrrole-4-carboxamide

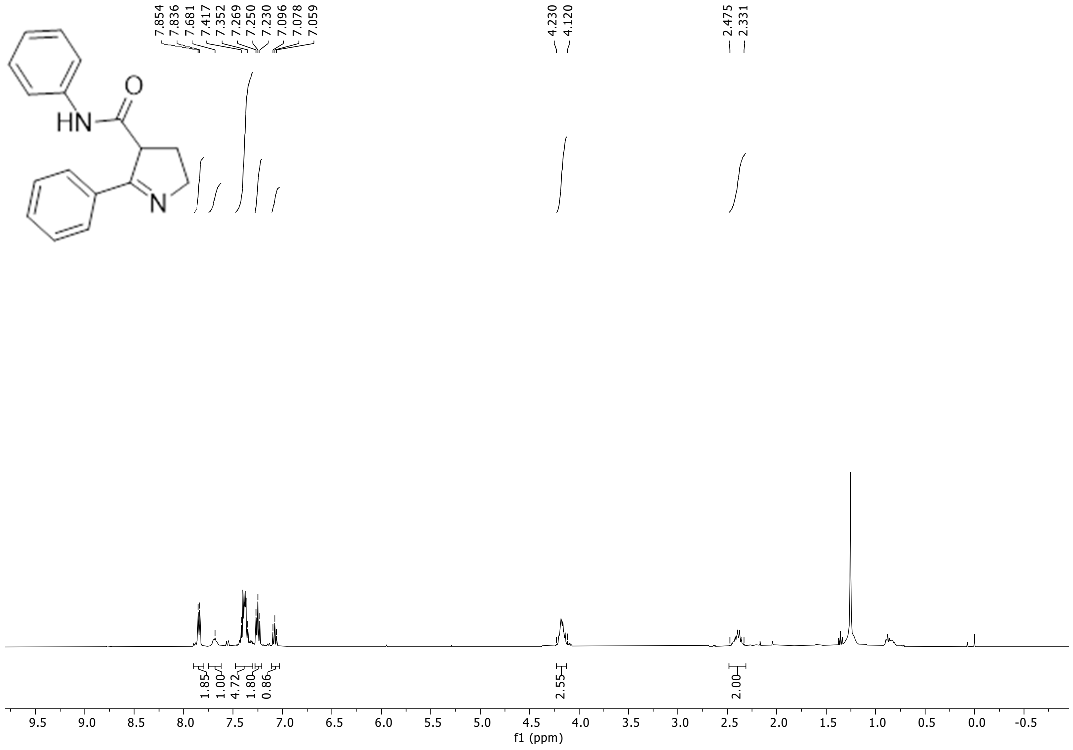

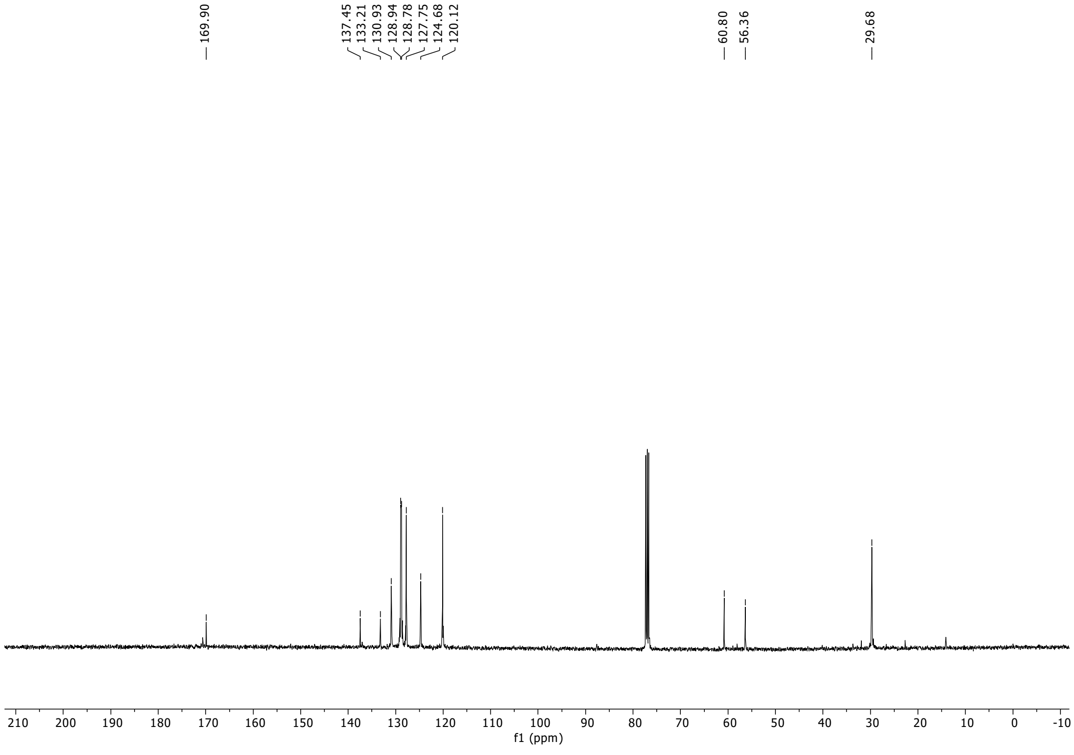

X-ray crystallographic data for 2d and 5

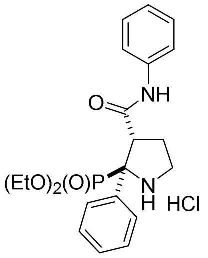

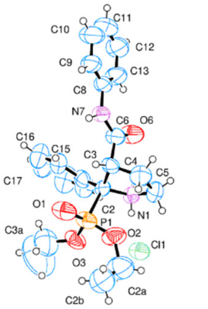

Diethyl[(2*RS*,3*SR*)-2-phenyl-3-(phenylcarbamoyl)pyrrolidin-2-yl]phosphonate hydrochloride, 2d

Table S1. Crystal data and structure refinement for **2d**

Identification code	**2d**	
Empirical formula	C21 H28 N2 O4 P Cl	
Formula weight	438.87	
Temperature	294(2) K	
Wavelength	0.71073 Å	
Crystal system	Orthorhombic	
Space group	P c a 21	
Unit cell dimensions	a = 12.645(4) Å	α= 90°.
	b = 13.977(5) Å	β= 90°.
	c = 25.770(9) Å	γ = 90°.
Volume	4555(3) Å^3	
Z	8	
Density (calculated)	1.280 Mg/m^3	
Absorption coefficient	0.266 mm^{-1}	
F(000)	1856	
Crystal size	0.250 x 0.240 x 0.080 mm^3	
Theta range for data collection	1.457 to 28.308°.	
Index ranges	-16<=h<=16, -18<=k<=18, -34<=l<=34	
Reflections collected	126927	
Independent reflections	11298 [R(int) = 0.1701]	
Completeness to theta = 25.242°	100.0 %	
Absorption correction	Semi-empirical from equivalents	
Max. and min. transmission	1 and 0.839	
Refinement method	Full-matrix least-squares on F^2	
Data / restraints / parameters	11298 / 7 / 527	
Goodness-of-fit on F^2	0.852	
Final R indices [I>2sigma(I)]	R1 = 0.0593, wR2 = 0.1277	
R indices (all data)	R1 = 0.1896, wR2 = 0.1732	
Absolute structure parameter	0.07(7)	
Largest diff. peak and hole	0.254 and -0.198 e.Å^{-3}	

Table S2. Atomic coordinates (x 10^4) and equivalent isotropic displacement parameters ($Å^2$x 10^3) For **2d**. U(eq) is defined as one third of the trace of the orthogonalized U^{ij} tensor.

	x	y	z	U(eq)
Cl(1)	5133(1)	7124(1)	3576(1)	92(1)
Cl(2)	7552(2)	12109(1)	6434(1)	96(1)
P(1)	2162(1)	5871(1)	3574(1)	81(1)
P(2)	4632(2)	10799(2)	6362(2)	92(1)
O(1)	1090(3)	5503(4)	3620(4)	109(2)
O(2)	2475(6)	6340(5)	3042(3)	105(2)
O(3)	2469(5)	6616(4)	3991(3)	98(2)
O(4)	3561(4)	10443(4)	6304(4)	121(3)
O(5)	4990(5)	11501(5)	5954(4)	119(3)
O(6)	4225(4)	3063(4)	3434(3)	115(3)
O(7)	4945(6)	11269(6)	6894(4)	133(3)
O(26)	6668(4)	7983(4)	6573(3)	95(2)
N(1)	4156(5)	5121(4)	3382(3)	63(2)
N(7)	2618(4)	2367(4)	3472(3)	70(2)
N(21)	6614(6)	10097(4)	6580(4)	71(2)
N(27)	5023(4)	7327(4)	6536(3)	67(2)
C(2)	3104(5)	4860(5)	3612(4)	56(2)
C(2A)	2437(10)	7327(9)	2858(6)	146(5)
C(2B)	1400(10)	7634(9)	2814(5)	133(4)
C(3)	2719(6)	4047(5)	3262(4)	65(2)
C(3A)	1901(13)	6750(11)	4466(7)	166(6)
C(3B)	2270(20)	7330(18)	4810(10)	334(17)
C(4)	3120(7)	4339(7)	2720(4)	91(3)
C(5)	4126(8)	4934(7)	2812(5)	84(4)
C(5A)	4350(20)	11880(20)	5568(10)	249(12)
C(5B)	4464(17)	11568(19)	5132(9)	235(11)
C(6)	3266(6)	3117(5)	3396(4)	76(3)
C(6A)	4678(17)	12204(12)	7058(11)	305(16)
C(6B)	3865(11)	12532(11)	7214(6)	173(6)
C(8)	2896(6)	1392(5)	3560(4)	71(2)
C(9)	2130(7)	725(6)	3506(5)	92(3)
C(10)	2303(10)	-209(7)	3580(6)	117(4)
C(11)	3335(11)	-505(8)	3713(5)	124(4)

C(12)	4112(9)	163(8)	3769(6)	114(5)
C(13)	3900(7)	1111(6)	3697(4)	102(3)
C(14)	3204(6)	4626(6)	4174(4)	71(2)
C(15)	2404(7)	4099(7)	4415(4)	85(3)
C(16)	2444(11)	3908(8)	4937(5)	119(4)
C(17)	3230(11)	4241(10)	5223(5)	132(4)
C(18)	4033(10)	4782(10)	5000(6)	130(5)
C(19)	3987(9)	4964(7)	4485(6)	89(4)
C(22)	5571(5)	9809(5)	6346(4)	61(3)
C(23)	5197(6)	9005(6)	6735(4)	75(3)
C(24)	5587(7)	9352(6)	7261(4)	90(3)
C(25)	6584(9)	9925(7)	7143(6)	93(4)
C(26)	5699(6)	8058(6)	6588(3)	68(2)
C(28)	5246(6)	6345(5)	6454(3)	72(2)
C(29)	4421(7)	5732(6)	6531(4)	88(3)
C(30)	4612(10)	4754(8)	6474(5)	104(4)
C(31)	5587(9)	4425(7)	6333(5)	106(3)
C(32)	6368(10)	5030(6)	6259(6)	99(4)
C(33)	6239(7)	6015(6)	6324(4)	92(3)
C(34)	5689(6)	9478(6)	5790(4)	73(2)
C(35)	4962(7)	8873(7)	5568(5)	92(3)
C(36)	5046(11)	8545(8)	5062(6)	116(4)
C(37)	5910(13)	8849(10)	4777(5)	132(4)
C(38)	6633(9)	9452(11)	4975(7)	126(5)
C(39)	6558(9)	9791(9)	5481(6)	95(4)

Table S3. Bond lengths [Å] and angles [°] for **2d**.

P(1)-O(1)	1.455(5)
P(1)-O(3)	1.546(7)
P(1)-O(2)	1.570(8)
P(1)-C(2)	1.852(7)
P(2)-O(4)	1.450(5)
P(2)-O(5)	1.508(8)
P(2)-O(7)	1.572(9)
P(2)-C(22)	1.824(7)
O(2)-C(2A)	1.459(13)
O(3)-C(3A)	1.431(16)
O(5)-C(5A)	1.38(3)
O(6)-C(6)	1.219(8)
O(7)-C(6A)	1.414(16)
O(26)-C(26)	1.231(8)
N(1)-C(5)	1.492(14)
N(1)-C(2)	1.503(10)
N(7)-C(6)	1.345(9)
N(7)-C(8)	1.426(9)
N(21)-C(25)	1.470(15)
N(21)-C(22)	1.505(11)
N(27)-C(26)	1.338(9)
N(27)-C(28)	1.417(9)
C(2)-C(14)	1.490(13)
C(2)-C(3)	1.530(12)
C(2A)-C(2B)	1.385(14)
C(3)-C(6)	1.514(10)
C(3)-C(4)	1.542(12)
C(3A)-C(3B)	1.29(2)
C(4)-C(5)	1.537(13)
C(5A)-C(5B)	1.21(3)
C(6A)-C(6B)	1.195(16)
C(8)-C(9)	1.351(11)
C(8)-C(13)	1.375(10)
C(9)-C(10)	1.338(12)
C(10)-C(11)	1.412(14)
C(11)-C(12)	1.363(15)

C(12)-C(13)	1.364(13)
C(14)-C(19)	1.357(13)
C(14)-C(15)	1.397(12)
C(15)-C(16)	1.373(14)
C(16)-C(17)	1.321(15)
C(17)-C(18)	1.390(16)
C(18)-C(19)	1.352(16)
C(22)-C(34)	1.513(14)
C(22)-C(23)	1.578(12)
C(23)-C(26)	1.517(11)
C(23)-C(24)	1.522(13)
C(24)-C(25)	1.524(13)
C(28)-C(29)	1.365(10)
C(28)-C(33)	1.378(10)
C(29)-C(30)	1.395(12)
C(30)-C(31)	1.365(13)
C(31)-C(32)	1.314(13)
C(32)-C(33)	1.397(11)
C(34)-C(35)	1.374(12)
C(34)-C(39)	1.426(14)
C(35)-C(36)	1.388(15)
C(36)-C(37)	1.383(17)
C(37)-C(38)	1.343(17)
C(38)-C(39)	1.390(18)
O(1)-P(1)-O(3)	114.6(4)
O(1)-P(1)-O(2)	117.0(5)
O(3)-P(1)-O(2)	105.3(4)
O(1)-P(1)-C(2)	108.9(3)
O(3)-P(1)-C(2)	108.4(4)
O(2)-P(1)-C(2)	101.7(4)
O(4)-P(2)-O(5)	115.6(5)
O(4)-P(2)-O(7)	117.9(5)
O(5)-P(2)-O(7)	105.1(5)
O(4)-P(2)-C(22)	110.2(4)
O(5)-P(2)-C(22)	106.5(4)
O(7)-P(2)-C(22)	100.0(5)
C(2A)-O(2)-P(1)	132.1(8)
C(3A)-O(3)-P(1)	123.8(7)

C(5A)-O(5)-P(2)	125.1(11)
C(6A)-O(7)-P(2)	125.9(14)
C(5)-N(1)-C(2)	108.8(8)
C(6)-N(7)-C(8)	128.1(6)
C(25)-N(21)-C(22)	109.2(8)
C(26)-N(27)-C(28)	128.8(6)
C(14)-C(2)-N(1)	111.2(7)
C(14)-C(2)-C(3)	116.0(7)
N(1)-C(2)-C(3)	103.3(8)
C(14)-C(2)-P(1)	105.9(6)
N(1)-C(2)-P(1)	111.3(5)
C(3)-C(2)-P(1)	109.3(6)
C(2B)-C(2A)-O(2)	110.5(11)
C(6)-C(3)-C(2)	111.0(7)
C(6)-C(3)-C(4)	106.5(7)
C(2)-C(3)-C(4)	103.5(7)
C(3B)-C(3A)-O(3)	119.4(16)
C(5)-C(4)-C(3)	106.0(8)
N(1)-C(5)-C(4)	105.6(8)
C(5B)-C(5A)-O(5)	118(3)
O(6)-C(6)-N(7)	123.2(7)
O(6)-C(6)-C(3)	121.8(7)
N(7)-C(6)-C(3)	115.1(6)
C(6B)-C(6A)-O(7)	131.2(19)
C(9)-C(8)-C(13)	119.4(7)
C(9)-C(8)-N(7)	117.8(7)
C(13)-C(8)-N(7)	122.8(7)
C(10)-C(9)-C(8)	122.8(9)
C(9)-C(10)-C(11)	118.1(10)
C(12)-C(11)-C(10)	119.5(10)
C(11)-C(12)-C(13)	120.6(11)
C(12)-C(13)-C(8)	119.6(10)
C(19)-C(14)-C(15)	116.7(10)
C(19)-C(14)-C(2)	124.0(9)
C(15)-C(14)-C(2)	119.0(8)
C(16)-C(15)-C(14)	120.7(10)
C(17)-C(16)-C(15)	120.3(11)
C(16)-C(17)-C(18)	120.7(13)

C(19)-C(18)-C(17)	118.5(12)
C(18)-C(19)-C(14)	123.0(12)
N(21)-C(22)-C(34)	112.0(7)
N(21)-C(22)-C(23)	101.4(8)
C(34)-C(22)-C(23)	114.5(7)
N(21)-C(22)-P(2)	111.0(5)
C(34)-C(22)-P(2)	108.5(7)
C(23)-C(22)-P(2)	109.3(5)
C(26)-C(23)-C(24)	111.4(7)
C(26)-C(23)-C(22)	109.7(7)
C(24)-C(23)-C(22)	104.0(7)
C(23)-C(24)-C(25)	104.9(9)
N(21)-C(25)-C(24)	107.8(9)
O(26)-C(26)-N(27)	124.6(7)
O(26)-C(26)-C(23)	120.0(7)
N(27)-C(26)-C(23)	115.1(6)
C(29)-C(28)-C(33)	121.4(7)
C(29)-C(28)-N(27)	115.8(6)
C(33)-C(28)-N(27)	122.8(7)
C(28)-C(29)-C(30)	117.9(8)
C(31)-C(30)-C(29)	121.0(10)
C(32)-C(31)-C(30)	120.0(10)
C(31)-C(32)-C(33)	122.0(10)
C(28)-C(33)-C(32)	117.7(9)
C(35)-C(34)-C(39)	118.2(11)
C(35)-C(34)-C(22)	121.0(8)
C(39)-C(34)-C(22)	120.8(9)
C(34)-C(35)-C(36)	122.9(10)
C(37)-C(36)-C(35)	117.2(12)
C(38)-C(37)-C(36)	122.0(14)
C(37)-C(38)-C(39)	121.5(13)
C(38)-C(39)-C(34)	118.2(12)

Table S4. Anisotropic displacement parameters ($Å^2$x 10^3) for **2d**. The anisotropic displacement factor exponent takes the form: $-2\pi^2[h^2a^{*2}U^{11} + ... + 2 h k a^* b^* U^{12}]$

	U^{11}	U^{22}	U^{33}	U^{23}	U^{13}	U^{12}
Cl(1)	46(1)	73(1)	158(2)	-3(2)	1(1)	-4(1)
Cl(2)	49(1)	75(1)	166(2)	-6(2)	4(1)	-4(1)
P(1)	33(1)	64(1)	145(2)	-7(2)	-8(1)	3(1)
P(2)	32(1)	61(1)	182(3)	17(2)	14(2)	3(1)
O(1)	31(2)	71(3)	225(7)	-10(5)	-2(4)	-1(2)
O(2)	93(5)	77(4)	147(7)	37(4)	-10(4)	13(4)
O(3)	57(3)	74(3)	162(7)	-33(4)	12(4)	2(3)
O(4)	27(2)	86(4)	251(8)	24(5)	14(4)	4(3)
O(5)	63(4)	85(4)	208(9)	59(5)	-18(5)	4(3)
O(6)	41(3)	79(4)	224(8)	10(5)	-4(4)	0(3)
O(7)	101(5)	99(5)	200(9)	-48(6)	15(6)	9(5)
O(26)	45(3)	72(3)	169(6)	-2(4)	-8(4)	7(2)
N(1)	31(4)	80(4)	77(7)	2(4)	2(4)	-5(3)
N(7)	47(3)	56(4)	105(6)	-1(4)	0(4)	1(3)
N(21)	39(4)	73(5)	100(8)	-6(4)	7(5)	-7(3)
N(27)	41(3)	61(4)	99(5)	6(4)	1(3)	0(3)
C(2)	30(4)	55(4)	82(7)	7(5)	3(5)	-2(3)
C(2A)	100(9)	132(10)	207(15)	29(10)	-14(8)	23(8)
C(2B)	116(9)	140(10)	143(10)	18(9)	-1(8)	7(8)
C(3)	44(4)	60(5)	90(6)	-2(5)	-4(4)	11(4)
C(3A)	159(13)	163(13)	177(15)	-71(11)	23(12)	-36(11)
C(3B)	310(30)	380(30)	300(30)	-220(30)	60(20)	-90(30)
C(4)	83(7)	92(7)	99(8)	-2(6)	-6(6)	-5(5)
C(5)	66(7)	97(8)	89(10)	16(5)	10(7)	-2(5)
C(5A)	240(30)	290(30)	210(20)	10(20)	-20(20)	-110(20)
C(5B)	177(18)	310(30)	220(20)	-80(20)	-22(18)	-16(16)
C(6)	48(4)	62(5)	119(8)	-5(5)	1(5)	-2(4)
C(6A)	270(20)	134(13)	510(40)	-57(18)	210(30)	60(14)
C(6B)	140(11)	182(14)	198(16)	-61(14)	40(12)	20(11)
C(8)	61(4)	60(4)	92(6)	4(5)	3(5)	12(4)
C(9)	72(5)	68(6)	136(8)	9(6)	7(6)	7(5)
C(10)	115(9)	71(6)	167(12)	-6(8)	4(9)	-4(6)
C(11)	134(10)	78(7)	160(12)	10(7)	13(9)	35(7)

C(12)	79(7)	93(7)	171(14)	16(7)	4(7)	22(6)
C(13)	70(5)	75(6)	161(10)	33(7)	4(6)	17(4)
C(14)	48(5)	72(5)	92(8)	-14(5)	8(5)	-1(4)
C(15)	70(6)	105(7)	81(8)	6(6)	15(5)	2(5)
C(16)	115(10)	145(10)	96(10)	8(8)	19(7)	-28(8)
C(17)	127(10)	170(12)	99(10)	6(9)	8(9)	-9(9)
C(18)	101(10)	183(13)	105(11)	-4(9)	-18(8)	-27(9)
C(19)	52(6)	115(9)	99(11)	3(6)	-12(7)	-6(5)
C(22)	27(4)	58(4)	99(9)	7(5)	9(5)	-2(3)
C(23)	45(4)	68(5)	113(8)	9(6)	16(5)	-2(4)
C(24)	91(7)	83(6)	96(8)	-6(6)	2(6)	-2(5)
C(25)	63(7)	93(8)	123(13)	-23(6)	-5(8)	-2(5)
C(26)	46(4)	66(5)	92(7)	6(4)	0(4)	1(4)
C(28)	58(4)	60(5)	97(7)	-1(5)	0(5)	8(4)
C(29)	64(5)	59(5)	142(9)	-3(6)	-7(5)	0(4)
C(30)	103(8)	81(7)	126(10)	-9(7)	-6(7)	-6(6)
C(31)	112(8)	69(6)	138(9)	-2(7)	-13(8)	9(6)
C(32)	87(8)	76(7)	134(10)	1(6)	18(8)	28(5)
C(33)	80(6)	80(6)	116(8)	2(6)	25(6)	12(5)
C(34)	40(5)	82(6)	97(8)	18(6)	-3(5)	1(4)
C(35)	76(6)	101(7)	98(9)	23(7)	-6(6)	-16(6)
C(36)	138(11)	109(8)	102(10)	-9(8)	-37(9)	-6(8)
C(37)	135(12)	164(13)	97(10)	17(9)	7(10)	35(10)
C(38)	80(8)	162(12)	137(15)	35(11)	10(8)	16(8)
C(39)	67(7)	127(9)	90(10)	9(7)	11(7)	13(6)

Table S5. Hydrogen bonds for **2d** [Å and °].

D-H...A	d(D-H)	d(H...A)	d(D...A)	<(DHA)
N(1)-H(1)...Cl(1)	0.86	2.55	3.100(7)	122.5
N(1)-H(1)...O(1)#1	0.86	2.14	2.668(8)	119.5
N(7)-H(7)...Cl(1)#2	0.86	2.38	3.232(6)	172.4
N(21)-H(21)...Cl(2)	0.86	2.55	3.075(7)	120.7
N(21)-H(21)...O(4)#3	0.86	2.11	2.671(9)	122.6
N(27)-H(27)...Cl(2)#4	0.86	2.38	3.233(6)	169.4
C(2A)-H(2A2)...Cl(2)#5	0.97	2.93	3.754(16)	143.4
C(3)-H(3)...Cl(1)#2	0.98	2.88	3.744(8)	148.1
C(3)-H(3)...O(1)	0.98	2.55	3.037(9)	110.8
C(6B)-H(6B1)...O(26)#4	0.96	2.48	3.310(16)	144.7
C(13)-H(13)...O(6)	0.93	2.25	2.842(10)	120.5
C(23)-H(23)...Cl(2)#4	0.98	2.90	3.770(9)	148.6
C(23)-H(23)...O(4)	0.98	2.60	3.091(10)	111.1
C(33)-H(33)...O(26)	0.93	2.30	2.875(11)	119.8

Symmetry transformations used to generate equivalent atoms:

#1 x+1/2,-y+1,z #2 x-1/2,-y+1,z #3 x+1/2,-y+2,z

#4 x-1/2,-y+2,z #5 -x+1,-y+2,z-1/2

Methyl (3RS,4SR,5SR)-4-((3-chloro-4-fluorophenyl)carbamoyl)-5-(diethoxyphosphoryl)-5-phenylpyrrolidine-3-carboxylate, 5

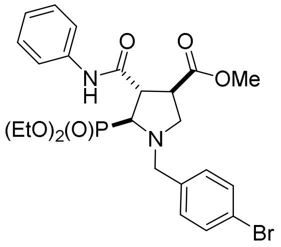

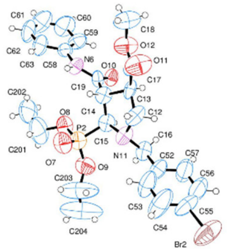

Table S6. Crystal data and structure refinement for **5**

Identification code	**5**	
Empirical formula	C24 H30 Br N2 O6 P	
Formula weight	553.38	
Temperature	293(2) K	
Wavelength	0.71069 Å	
Crystal system	Monoclinic	
Space group	C 2/c	
Unit cell dimensions	a = 44.05(2) Å	α= 90°.
	b = 10.843(7) Å	β= 95.67(5)°.
	c = 22.619(9) Å	γ = 90°.
Volume	10751(10) $Å^3$	
Z	16	
Density (calculated)	1.368 Mg/m^3	
Absorption coefficient	1.628 mm^{-1}	
F(000)	4576	
Crystal size	0.33 x 0.24 x 0.06 mm^3	
Theta range for data collection	1.809 to 25.037°.	
Index ranges	-52<=h<=52, -12<=k<=12, -26<=l<=0	
Reflections collected	19652	
Independent reflections	9467 [R(int) = 0.0557]	
Completeness to theta = 25.037°	99.6 %	
Refinement method	Full-matrix least-squares on F^2	
Data / restraints / parameters	9467 / 196 / 619	
Goodness-of-fit on F^2	0.790	
Final R indices [I>2sigma(I)]	R1 = 0.0772, wR2 = 0.0918	
R indices (all data)	R1 = 0.3261, wR2 = 0.1418	
Largest diff. peak and hole	0.235 and -0.259 e.$Å^{-3}$	

Table S7. Atomic coordinates (x 10^4) and equivalent isotropic displacement parameters (Å^{2}x 10^3) For **5**. U(eq) is defined as one third of the trace of the orthogonalized U^{ij} tensor.

	x	y	z	U(eq)
Br(1)	7129(1)	-4945(2)	-1188(1)	151(1)
Br(2)	5353(1)	-4850(2)	-3069(1)	169(1)
P(1)	6515(1)	-7765(3)	-4675(1)	72(1)
P(2)	5967(1)	-7855(4)	482(1)	81(1)
O(1)	6590(2)	-8995(6)	-4428(3)	88(2)
O(2)	6422(2)	-7713(7)	-5361(3)	102(3)
O(3)	6783(2)	-6804(7)	-4583(3)	93(2)
O(4)	5639(2)	-6671(6)	-5226(3)	89(3)
O(5)	5495(2)	-8641(10)	-3296(3)	180(5)
O(6)	5287(2)	-8381(10)	-4170(3)	152(4)
O(7)	5865(2)	-9070(6)	242(3)	99(2)
O(8)	6114(2)	-7876(6)	1150(3)	89(2)
O(9)	5709(2)	-6859(7)	455(3)	104(2)
O(10)	6869(2)	-6693(6)	854(3)	71(2)
O(11)	6929(2)	-8794(8)	-1090(3)	123(3)
O(12)	7172(2)	-8405(8)	-224(3)	113(3)
N(1)	6273(2)	-6796(8)	-3742(3)	67(2)
N(2)	5706(2)	-8723(7)	-5380(3)	77(3)
N(6)	6833(2)	-8751(7)	1048(3)	66(3)
N(11)	6171(2)	-6940(7)	-500(3)	71(2)
C(2)	6002(2)	-7128(9)	-3454(4)	80(3)
C(3)	5737(2)	-7360(9)	-3931(4)	73(3)
C(4)	5916(2)	-7833(9)	-4452(4)	55(2)
C(5)	6198(2)	-7022(8)	-4376(4)	57(2)
C(6)	6383(2)	-5550(8)	-3621(4)	80(3)
C(7)	5497(3)	-8230(13)	-3760(5)	88(4)
C(8)	5051(3)	-9223(12)	-4047(5)	205(8)
C(9)	5728(3)	-7671(10)	-5051(4)	64(3)
C(12)	6410(2)	-7399(9)	-851(4)	90(4)
C(13)	6707(2)	-7510(8)	-418(4)	65(3)
C(14)	6565(2)	-7948(9)	150(4)	68(3)
C(15)	6277(2)	-7149(9)	137(4)	69(3)
C(16)	6080(2)	-5662(8)	-639(4)	76(3)

C(17)	6940(3)	-8311(11)	-631(5)	77(4)
C(18)	7426(2)	-9185(11)	-369(4)	135(5)
C(19)	6770(2)	-7707(10)	725(4)	64(3)
C(40)	6550(2)	-5436(9)	-3005(4)	64(3)
C(41)	6640(2)	-6441(9)	-2662(4)	77(4)
C(42)	6811(2)	-6302(10)	-2122(4)	77(4)
C(43)	6891(2)	-5160(13)	-1934(4)	73(3)
C(44)	6818(3)	-4112(10)	-2266(4)	104(5)
C(45)	6642(2)	-4281(9)	-2813(4)	84(4)
C(46)	5601(2)	-8865(10)	-5993(4)	61(3)
C(47)	5426(2)	-7937(10)	-6298(4)	92(4)
C(48)	5326(3)	-8156(12)	-6891(5)	120(5)
C(49)	5389(3)	-9234(12)	-7162(4)	99(5)
C(50)	5562(2)	-10174(12)	-6848(4)	103(4)
C(51)	5662(2)	-9955(11)	-6259(4)	79(3)
C(52)	5904(3)	-5511(11)	-1242(5)	83(4)
C(53)	5690(3)	-6274(11)	-1470(5)	128(5)
C(54)	5508(4)	-6115(13)	-2007(6)	195(9)
C(55)	5587(4)	-5169(16)	-2329(5)	120(6)
C(56)	5807(4)	-4454(14)	-2139(6)	153(7)
C(57)	5989(3)	-4612(11)	-1612(5)	131(5)
C(58)	7015(2)	-8862(11)	1604(4)	67(3)
C(59)	7205(2)	-7980(11)	1846(4)	99(4)
C(60)	7374(3)	-8231(12)	2396(5)	127(5)
C(61)	7326(3)	-9280(14)	2698(6)	138(6)
C(62)	7123(3)	-10146(12)	2453(5)	114(5)
C(63)	6970(2)	-9970(11)	1905(4)	95(4)
C(101)	6597(4)	-8351(14)	-5776(5)	181(8)
C(102)	6466(3)	-8540(13)	-6307(4)	192(8)
C(103)	7058(3)	-6879(13)	-4118(6)	139(6)
C(104)	7281(3)	-7445(15)	-4278(7)	226(9)
C(201)	5966(3)	-8574(13)	1591(5)	162(7)
C(202)	6166(3)	-8918(12)	2075(4)	165(6)
C(203)	5397(4)	-6994(18)	287(10)	248(11)
C(204)	5178(3)	-6362(18)	275(10)	290(12)

Table S8. Bond lengths [Å] and angles [°] for **5**

Br(1)-C(43)	1.912(8)
Br(2)-C(55)	1.910(11)
P(1)-O(1)	1.471(6)
P(1)-O(2)	1.565(6)
P(1)-O(3)	1.574(7)
P(1)-C(5)	1.802(9)
P(2)-O(7)	1.477(6)
P(2)-O(9)	1.566(8)
P(2)-O(8)	1.584(6)
P(2)-C(15)	1.808(10)
O(2)-C(101)	1.448(12)
O(3)-C(103)	1.527(11)
O(4)-C(9)	1.206(10)
O(5)-C(7)	1.140(11)
O(6)-C(7)	1.256(11)
O(6)-C(8)	1.431(11)
O(8)-C(201)	1.456(12)
O(9)-C(203)	1.394(14)
O(10)-C(19)	1.207(10)
O(11)-C(17)	1.160(10)
O(12)-C(17)	1.309(11)
O(12)-C(18)	1.465(10)
N(1)-C(6)	1.452(9)
N(1)-C(2)	1.460(10)
N(1)-C(5)	1.461(9)
N(2)-C(9)	1.359(10)
N(2)-C(46)	1.426(9)
N(6)-C(19)	1.361(10)
N(6)-C(58)	1.427(10)
N(11)-C(12)	1.466(11)
N(11)-C(16)	1.468(10)
N(11)-C(15)	1.487(9)
C(2)-C(3)	1.531(10)
C(3)-C(7)	1.499(13)
C(3)-C(4)	1.566(11)
C(4)-C(5)	1.518(11)

C(4)-C(9)	1.526(11)
C(6)-C(40)	1.515(10)
C(12)-C(13)	1.561(11)
C(13)-C(17)	1.460(13)
C(13)-C(14)	1.558(10)
C(14)-C(19)	1.531(11)
C(14)-C(15)	1.535(11)
C(16)-C(52)	1.509(11)
C(40)-C(41)	1.372(10)
C(40)-C(45)	1.373(11)
C(41)-C(42)	1.377(10)
C(42)-C(43)	1.345(12)
C(43)-C(44)	1.382(12)
C(44)-C(45)	1.407(10)
C(46)-C(51)	1.364(12)
C(46)-C(47)	1.407(11)
C(47)-C(48)	1.390(10)
C(48)-C(49)	1.362(14)
C(49)-C(50)	1.418(13)
C(50)-C(51)	1.382(10)
C(52)-C(53)	1.323(13)
C(52)-C(57)	1.361(13)
C(53)-C(54)	1.396(12)
C(54)-C(55)	1.324(17)
C(55)-C(56)	1.282(16)
C(56)-C(57)	1.378(13)
C(58)-C(59)	1.350(12)
C(58)-C(63)	1.405(12)
C(59)-C(60)	1.412(11)
C(60)-C(61)	1.355(14)
C(61)-C(62)	1.374(14)
C(62)-C(63)	1.366(11)
C(101)-C(102)	1.298(12)
C(103)-C(104)	1.242(13)
C(201)-C(202)	1.389(12)
C(203)-C(204)	1.181(18)
O(1)-P(1)-O(2)	115.9(4)

O(1)-P(1)-O(3)	114.4(4)
O(2)-P(1)-O(3)	103.1(4)
O(1)-P(1)-C(5)	114.6(4)
O(2)-P(1)-C(5)	102.6(4)
O(3)-P(1)-C(5)	104.8(4)
O(7)-P(2)-O(9)	114.0(5)
O(7)-P(2)-O(8)	114.7(4)
O(9)-P(2)-O(8)	105.9(4)
O(7)-P(2)-C(15)	115.7(5)
O(9)-P(2)-C(15)	105.5(5)
O(8)-P(2)-C(15)	99.4(4)
C(101)-O(2)-P(1)	121.4(8)
C(103)-O(3)-P(1)	126.1(8)
C(7)-O(6)-C(8)	115.8(11)
C(201)-O(8)-P(2)	119.5(7)
C(203)-O(9)-P(2)	129.0(10)
C(17)-O(12)-C(18)	116.6(9)
C(6)-N(1)-C(2)	114.7(8)
C(6)-N(1)-C(5)	112.4(7)
C(2)-N(1)-C(5)	106.3(8)
C(9)-N(2)-C(46)	128.5(9)
C(19)-N(6)-C(58)	127.6(9)
C(12)-N(11)-C(16)	113.5(8)
C(12)-N(11)-C(15)	107.5(8)
C(16)-N(11)-C(15)	113.8(8)
N(1)-C(2)-C(3)	108.9(8)
C(7)-C(3)-C(2)	115.4(9)
C(7)-C(3)-C(4)	114.1(9)
C(2)-C(3)-C(4)	100.4(8)
C(5)-C(4)-C(9)	113.5(8)
C(5)-C(4)-C(3)	101.0(8)
C(9)-C(4)-C(3)	111.3(8)
N(1)-C(5)-C(4)	108.2(8)
N(1)-C(5)-P(1)	109.5(6)
C(4)-C(5)-P(1)	110.8(7)
N(1)-C(6)-C(40)	111.8(8)
O(5)-C(7)-O(6)	123.8(14)
O(5)-C(7)-C(3)	123.8(13)

O(6)-C(7)-C(3)	112.2(11)
O(4)-C(9)-N(2)	124.8(10)
O(4)-C(9)-C(4)	121.6(10)
N(2)-C(9)-C(4)	113.2(9)
N(11)-C(12)-C(13)	106.8(8)
C(17)-C(13)-C(14)	116.3(9)
C(17)-C(13)-C(12)	114.4(9)
C(14)-C(13)-C(12)	99.2(8)
C(19)-C(14)-C(15)	109.8(8)
C(19)-C(14)-C(13)	113.3(8)
C(15)-C(14)-C(13)	102.2(8)
N(11)-C(15)-C(14)	106.5(8)
N(11)-C(15)-P(2)	107.8(7)
C(14)-C(15)-P(2)	114.3(7)
N(11)-C(16)-C(52)	113.6(8)
O(11)-C(17)-O(12)	123.7(13)
O(11)-C(17)-C(13)	126.5(13)
O(12)-C(17)-C(13)	109.8(10)
O(10)-C(19)-N(6)	125.3(10)
O(10)-C(19)-C(14)	121.9(10)
N(6)-C(19)-C(14)	112.7(10)
C(41)-C(40)-C(45)	119.1(10)
C(41)-C(40)-C(6)	122.8(9)
C(45)-C(40)-C(6)	117.7(9)
C(40)-C(41)-C(42)	121.1(10)
C(43)-C(42)-C(41)	118.9(10)
C(42)-C(43)-C(44)	123.1(9)
C(42)-C(43)-Br(1)	119.7(10)
C(44)-C(43)-Br(1)	117.2(9)
C(43)-C(44)-C(45)	116.7(10)
C(40)-C(45)-C(44)	121.0(10)
C(51)-C(46)-C(47)	121.7(10)
C(51)-C(46)-N(2)	117.7(10)
C(47)-C(46)-N(2)	120.5(10)
C(48)-C(47)-C(46)	117.2(11)
C(49)-C(48)-C(47)	121.4(13)
C(48)-C(49)-C(50)	120.9(11)
C(51)-C(50)-C(49)	117.6(12)

C(46)-C(51)-C(50)	121.1(11)
C(53)-C(52)-C(57)	115.9(13)
C(53)-C(52)-C(16)	124.6(12)
C(57)-C(52)-C(16)	119.2(13)
C(52)-C(53)-C(54)	126.0(14)
C(55)-C(54)-C(53)	114.5(15)
C(56)-C(55)-C(54)	121.0(14)
C(56)-C(55)-Br(2)	120.6(15)
C(54)-C(55)-Br(2)	118.3(14)
C(55)-C(56)-C(57)	124.7(15)
C(52)-C(57)-C(56)	116.8(13)
C(59)-C(58)-C(63)	121.4(11)
C(59)-C(58)-N(6)	124.7(11)
C(63)-C(58)-N(6)	113.8(11)
C(58)-C(59)-C(60)	118.2(12)
C(61)-C(60)-C(59)	120.7(13)
C(60)-C(61)-C(62)	119.9(13)
C(63)-C(62)-C(61)	120.9(13)
C(62)-C(63)-C(58)	118.6(12)
C(102)-C(101)-O(2)	117.3(14)
C(104)-C(103)-O(3)	114.9(15)
C(202)-C(201)-O(8)	112.7(12)
C(204)-C(203)-O(9)	136(2)

Table S9. Anisotropic displacement parameters ($Å^2$x 10^3) for **5**. The anisotropic displacement factor exponent takes the form: $-2\pi^2[h^2a^{*2}U^{11} + ... + 2 h k a^* b^* U^{12}]$

	U^{11}	U^{22}	U^{33}	U^{23}	U^{13}	U^{12}
Br(1)	180(2)	189(2)	76(1)	-12(1)	-29(1)	-56(2)
Br(2)	220(2)	204(2)	77(1)	14(1)	-12(1)	97(2)
P(1)	86(3)	70(3)	61(2)	1(2)	6(2)	3(2)
P(2)	94(3)	83(3)	67(2)	-13(2)	5(2)	-6(2)
O(1)	87(6)	66(5)	107(5)	5(4)	-2(4)	14(4)
O(2)	125(7)	114(7)	66(4)	-18(4)	-2(4)	12(5)
O(3)	95(6)	90(6)	95(5)	2(5)	4(4)	-26(5)
O(4)	132(7)	60(5)	68(5)	19(4)	-26(4)	3(5)
O(5)	215(11)	218(11)	101(7)	60(7)	-13(7)	-104(8)
O(6)	76(7)	257(12)	116(7)	52(7)	-18(5)	-67(7)
O(7)	117(7)	83(5)	97(5)	-21(4)	2(4)	-32(5)
O(8)	112(6)	94(6)	63(4)	-8(4)	15(4)	-19(5)
O(9)	93(6)	99(6)	121(6)	-15(5)	9(5)	-1(5)
O(10)	91(6)	50(5)	69(5)	3(4)	-9(4)	-17(5)
O(11)	139(7)	145(8)	83(6)	-47(5)	0(5)	44(6)
O(12)	104(7)	146(8)	85(6)	-27(6)	-4(5)	28(6)
N(1)	84(6)	73(7)	46(5)	5(5)	6(4)	-14(5)
N(2)	104(8)	59(6)	61(5)	0(5)	-19(5)	4(6)
N(6)	85(7)	44(5)	67(6)	-10(4)	-7(5)	2(5)
N(11)	114(7)	44(6)	53(5)	-18(4)	-3(4)	13(5)
C(2)	88(8)	93(9)	60(6)	-1(7)	11(5)	-14(7)
C(3)	87(7)	77(9)	55(6)	-10(6)	6(5)	-11(6)
C(4)	71(6)	41(7)	54(5)	8(5)	4(4)	2(5)
C(5)	93(6)	24(6)	53(5)	10(5)	-2(5)	-6(5)
C(6)	110(9)	72(8)	54(6)	-4(5)	-12(5)	-23(7)
C(7)	63(8)	137(13)	65(8)	13(8)	11(6)	-16(7)
C(8)	91(11)	340(20)	182(13)	71(13)	-19(9)	-106(11)
C(9)	88(9)	52(7)	50(6)	21(5)	1(5)	-19(7)
C(12)	143(9)	80(9)	48(6)	-5(6)	5(6)	39(8)
C(13)	114(7)	29(7)	54(6)	0(5)	11(5)	0(6)
C(14)	88(7)	67(8)	49(5)	-17(5)	5(4)	-1(6)
C(15)	95(6)	64(8)	46(5)	-20(5)	-4(5)	-10(5)
C(16)	114(10)	53(7)	63(6)	-6(5)	19(6)	1(6)

C(17)	120(9)	60(9)	52(7)	-6(7)	6(6)	21(7)
C(18)	112(11)	150(13)	139(11)	-23(9)	-10(8)	53(9)
C(19)	80(8)	53(7)	60(6)	5(6)	8(5)	-11(7)
C(40)	90(9)	40(7)	61(6)	2(5)	4(5)	-8(6)
C(41)	103(10)	59(7)	64(7)	-8(6)	-13(6)	17(7)
C(42)	97(10)	68(7)	66(7)	0(6)	0(6)	-9(7)
C(43)	74(8)	103(9)	44(6)	-8(6)	13(5)	-37(9)
C(44)	179(15)	66(8)	67(8)	-20(6)	9(7)	-54(9)
C(45)	125(11)	53(7)	71(7)	5(6)	-13(6)	-23(7)
C(46)	61(8)	71(8)	53(6)	0(5)	15(6)	-10(7)
C(47)	108(11)	89(9)	74(7)	1(7)	-22(7)	2(7)
C(48)	146(13)	127(12)	76(8)	8(8)	-35(9)	-26(10)
C(49)	95(11)	166(14)	36(7)	2(6)	0(6)	-41(10)
C(50)	89(10)	148(12)	75(7)	-49(8)	11(7)	-8(9)
C(51)	95(8)	89(8)	54(5)	2(6)	10(6)	-1(8)
C(52)	122(12)	58(9)	67(8)	0(6)	5(6)	19(7)
C(53)	193(17)	101(12)	82(9)	20(8)	-28(8)	-27(9)
C(54)	310(20)	133(15)	117(13)	25(9)	-106(13)	-28(13)
C(55)	155(16)	137(17)	62(8)	5(9)	-20(8)	81(11)
C(56)	200(20)	150(17)	107(12)	58(11)	-6(8)	28(11)
C(57)	157(13)	116(13)	115(10)	46(8)	-17(9)	-34(10)
C(58)	55(8)	83(9)	61(7)	-1(6)	5(5)	21(7)
C(59)	107(11)	94(9)	90(8)	-9(7)	-27(7)	10(7)
C(60)	137(13)	143(13)	89(10)	2(8)	-46(8)	-16(10)
C(61)	178(17)	139(15)	85(10)	3(8)	-48(9)	8(11)
C(62)	167(15)	106(12)	72(8)	37(8)	21(7)	0(9)
C(63)	131(10)	93(9)	60(7)	7(7)	4(6)	9(9)
C(101)	260(20)	187(17)	92(9)	-56(12)	17(11)	77(14)
C(102)	310(20)	185(15)	86(9)	-20(11)	26(11)	78(15)
C(103)	77(10)	167(16)	174(14)	5(12)	10(8)	11(9)
C(104)	135(13)	215(19)	340(20)	3(16)	80(12)	82(14)
C(201)	205(18)	201(18)	79(9)	11(10)	12(9)	-84(13)
C(202)	168(15)	223(16)	109(11)	65(10)	39(9)	-13(12)
C(203)	119(12)	250(20)	350(20)	-110(19)	-110(20)	50(13)
C(204)	113(13)	410(30)	330(20)	-110(20)	-57(18)	80(16)

Table S10. Hydrogen bonds for **5** [Å and °].

D-H...A	d(D-H)	d(H...A)	d(D...A)	<(DHA)
N(2)-H(2)...O(7)#1	0.86	1.97	2.830(9)	176.7
N(6)-H(6)...O(1)#2	0.86	1.98	2.837(9)	177.2
C(4)-H(4A)...O(7)#1	0.98	2.58	3.431(12)	144.7
C(6)-H(6B)...O(3)	0.97	2.56	3.232(12)	126.1
C(14)-H(14)...O(1)#2	0.98	2.59	3.448(12)	146.3
C(16)-H(16B)...O(4)#3	0.97	2.64	3.377(12)	133.3
C(18)-H(18B)...Br(1)#4	0.96	3.02	3.971(10)	170.9
C(42)-H(42)...O(11)	0.93	2.66	3.575(13)	166.6
C(47)-H(47)...O(4)	0.93	2.28	2.864(11)	120.1
C(59)-H(59)...O(10)	0.93	2.35	2.918(12)	119.5

Symmetry transformations used to generate equivalent atoms:

#1 x,-y-2,z-1/2 #2 x,-y-2,z+1/2 #3 x,-y-1,z+1/2

#4 -x+3/2,-y-3/2,-z

In vitro Blood-Brain Barrier Permeation Assay

Parallel Artificial Membrane Permeation Assays- Blood-Brain Barrier (PAMPA-BBB)

To evaluate the brain penetration of the different compounds, a parallel artificial membrane permeation assay for blood-brain barrier was used, following the method described by Di et al [1]. The in vitro permeability (Pe) of fourteen commercial drugs through lipid extract of porcine brain membrane together with the test compounds were determined. Commercial drugs and assayed compounds were tested using a mixture of PBS:ETOH (70:30). Assay validation was made by comparing the experimental permeability with the reported values of the commercial drugs by bibliography and lineal correlation between experimental and reported permeability of the fourteen commercial drugs using the parallel artificial membrane permeation assay was evaluated (y=1,584x-1,306; R2=0,9409). From this equation and taking into account the limits established by Di et al. for BBB permeation, we established the ranges of permeability as compounds of high BBB permeation (CNS+): Pe (10-6 cm s-1) > 5,030; compounds of low BBB permeation(CNS-): Pe (10-6 cm s-1) < 1,862 and compounds of uncertain BBB permeation(CNS+/-)<5,030 Pe (10-6 cm s-1) > 1,862.

Table S11 shows permeability results from the different commercial and assayed compounds (three different experiments in triplicate) and predictive penetration in the CNS.

Table S11. Permeability (*Pe* 10^{-6} cm s^{-1}) in the PAMPA-BBB assay of 14 commercial drugs and tested compounds and predictive penetration in the CNS.

Compound	Bibliography value[(a)]	Experimental value (n=3) ± S.D.	CNS Prediction
**Verapamil**	16,0	25,2±0,8	
**Testosterone**	17,0	28,5±0,6	
**Costicosterone**	5,1	6,7±0,1	
**Clonidine**	5,3	6,5±0,05	
**Ofloxacin**	0,8	1,1±0,06	
**Lomefloxacin**	0,0	0,8±0,02	
**Progesterone**	9,3	16,8±0,3	
**Promazine**	8,8	13,8±0,3	
**Imipramine**	13,0	12,5±0,2	
**Hidrocortisone**	1,9	1,4±0,05	
**Piroxicam**	2,5	2,4±0,07	
**Desipramine**	12,0	17,8±0,1	
**Cimetidine**	0,0	0,7±0,03	
**Norfloxacine**	0,1	0,8±0,05	
**2e**		14,6±0,4	CNS+
**4e**		14,35±0,6	CNS+

[a]Taken from Di et al. (2003).

Molecular Formula Strings (SMILES)

Table S12: Molecular Formula Strings of compounds **2a-g, 4a-e** and **5.**

Comp.	Structure	SMILE
**2a**	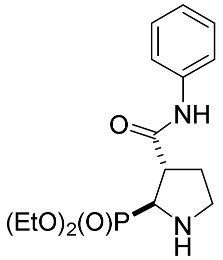	O=C([C@H]1[C@H](P(OCC)(OCC)=O)NCC1)NC2=CC=CC=C2
**2b**	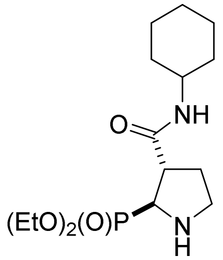	O=C([C@H]1[C@H](P(OCC)(OCC)=O)NCC1)NC2CCCCC2
**2c**	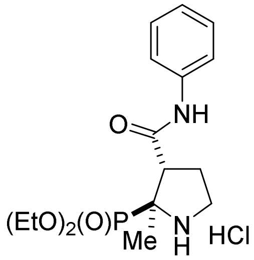	O=C([C@H]1[C@@](P(OCC)(OCC)=O)(C)NCC1)NC2=CC=CC=C2.Cl
**2d**	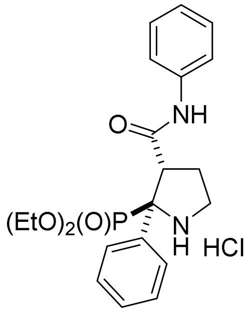	O=C([C@H]1[C@@](P(OCC)(OCC)=O)(C2=CC=CC=C2)NCC1)NC3=CC=CC=C3.Cl
**2e**	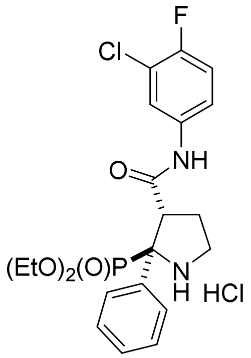	O=C([C@H]1[C@@](P(OCC)(OCC)=O)(C2=CC=CC=C2)NCC1)NC3=CC=C(F)C(Cl)=C3.Cl

**2f**	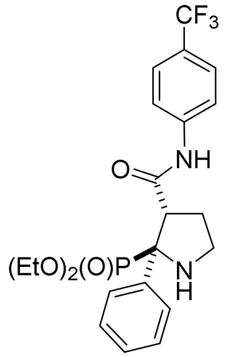	O=C([C@H]1[C@@](P(OCC)(OCC)=O)(C2=CC=CC=C2)NCC1)NC3=CC=C(C(F)(F)F)C=C3
**2g**	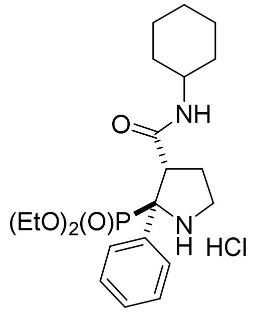	O=C([C@H]1[C@@](P(OCC)(OCC)=O)(C2=CC=CC=C2)NCC1)NC3CCCCC3.Cl
**4a**	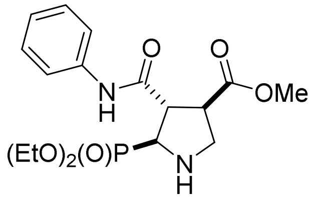	O=P(OCC)(OCC)[C@H]1[C@H](C(NC2=CC=CC=C2)=O)[C@@H](C(OC)=O)CN1
**4b**	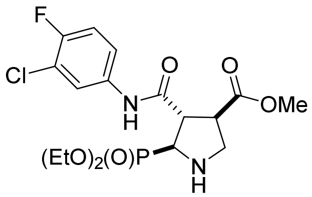	O=P(OCC)(OCC)[C@H]1[C@H](C(NC2=CC=C(F)C(Cl)=C2)=O)[C@@H](C(OC)=O)CN1
**4c**	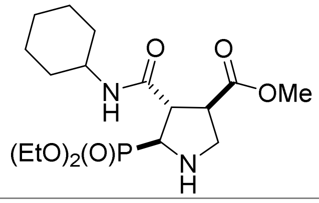	O=P(OCC)(OCC)[C@H]1[C@H](C(NC2CCCCC2)=O)[C@@H](C(OC)=O)CN1
**4d**	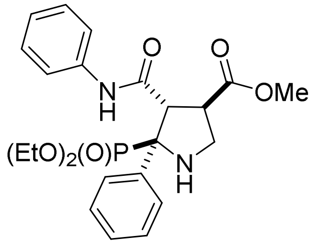	O=P(OCC)(OCC)[C@@]1(C2=CC=CC=C2)[C@H](C(NC3=CC=CC=C3)=O)[C@@H](C(OC)=O)CN1

**4e**	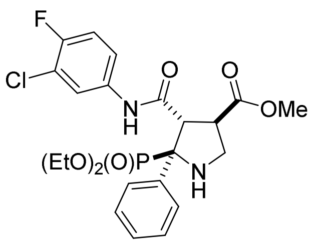	O=P(OCC)(OCC)[C@@]1(C2=CC=CC=C2)[C@H](C(NC3=CC=C(F)C(Cl)=C3)=O)[C@@H](C(OC)=O)CN1
**5**	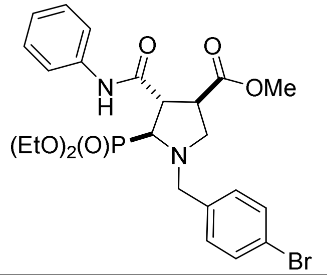	O=P(OCC)(OCC)[C@H]1[C@H](C(NC2=CC=CC=C2)=O)[C@@H](C(OC)=O)CN1CC3=CC=C(Br)C=C3

References

[1]. Di, L.; Kerns, E. H.; Fan, K.; McConnell, O. J.; CarTer, G. T. High throughput artificial membrane permeability assay for blood-brain barrier. *Eur. J. Med.Chem.* **2003**, *38*, 223-232.
